# Development of a Novel AAV-Mediated microRNA Gene Therapy for Spatial Suppression of BACE1 to Improve Cognitive Function in Alzheimer’s Disease Model Mice

**DOI:** 10.3390/biom16081075

**Published:** 2026-07-23

**Authors:** Ying Zhou, Yuelin Diao, Zhexiao Yan, Xindong Shui, Zichen Huang, Yan Sun, Siyao Wang, Yanqing Xia, Tae Ho Lee, Long Wang

**Affiliations:** 1 College of Biological Science and Engineering, Fuzhou University, Fuzhou 350108, China; 2 Fujian Key Laboratory of Cognitive Function and Diseases, Fujian Provincial Key Laboratory of Brain Aging and Neurodegenerative Diseases, Institute of Basic Medicine, School of Basic Medical Sciences, Fujian Medical University, Fuzhou 350122, China; 3 Key Laboratory of Non-Coding RNA and Drug Discovery, School of Basic Medical Sciences, Chengdu Medical College, Chengdu 610500, China; 4 School of Basic Medical Sciences, Ningxia Medical University, Yinchuan 750004, China

**Keywords:** Alzheimer’s disease, BACE1, adeno-associated virus, gene therapy, miRNA, cognitive function

## Abstract

The beta-site amyloid precursor protein (APP)-cleaving enzyme 1 (BACE1) is a promising and rational target for Alzheimer’s disease (AD), but current clinical trials have been disappointing. Consequently, utilizing the intrinsic regulatory mechanisms of BACE1 during AD pathogenesis might provide valuable insights into the treatment of this devastating disease. In this study, we proposed a combination of AAV delivery and microRNA therapeutics targeting AD at its root by sustained and spatial inhibition of BACE1 with a single therapeutic injection. We demonstrate that upregulation of BACE1 is correlated with downregulation of miR-143-3p in the hippocampus of individuals with AD, and miR-143-3p can directly target BACE1 to inhibit Aβ generation. In the brains of 5×FAD model mice, BACE1 levels are found to be elevated with age in the cornu ammonis 1 (CA1) subfield of the hippocampus. AAV-mediated miR-143-3p restoration in the hippocampal CA1 subfield of AD mice can improve cognitive performance, attenuate BACE1 expression, reduce Aβ levels, induce microglia polarization toward the anti-inflammatory phenotype, modulate neural-related genes including Gal3, and promote synaptic functions. Collectively, the AAV-mediated microRNA gene therapy approach developed for spatial suppression of BACE1 can effectively enhance cognitive performance in AD model mice, offering an attractive therapeutic option for AD treatment with long-lasting efficacy.

## 1. Introduction

With the global population aging and life expectancy increasing, the burden of Alzheimer’s disease (AD) on patients, caregivers, and society is rapidly escalating [1,2]. Pathologically, AD is characterized by the accumulation of amyloid plaques and neurofibrillary tangles in the cortex and hippocampus [3,4,5]. The recent encouraging results of anti-amyloid antibodies, including lecanemab [6,7], aducanumab [8,9], and donanemab [10], have strengthened the importance of the amyloid cascade hypothesis [11,12]. However, the clinical benefits of these immunotherapies targeting beta-amyloid (Aβ) only slow the progression of early AD instead of halting or reversing disease progression, and a series of side effects called amyloid-related imaging abnormalities (ARIA), such as brain swelling or bleeding have raised big concerns [2,11,12,13]. Therefore, a deeper understanding of Aβ pathologies in AD brains becomes particularly relevant.

The beta-site amyloid precursor protein (APP)-cleaving enzyme 1 (BACE1) is responsible for the first step of the amyloidogenic pathway, and this rate-limiting enzyme has long been considered a promising target for AD treatment [14,15,16,17]. Unexpectedly, to date, the clinical trials of BACE1 inhibitors LY2886721, verubecestat, atabecestat, lanabecestat, elenbecestat, and umibecestat have been disappointing, as a result of ineffectiveness or various adverse effects, such as cognitive worsening [12,16,18]. Interestingly, a recent study demonstrated that the cognitive decline due to umibecestat could be reversed shortly after treatment washout [19], indicating a critical role of BACE1 in the balance of Aβ production and cognitive performance. Considering the fact that physiological levels of BACE1 are essential for axonal organization, synaptic function, and cognitive circuit [20,21,22], it is of significance to seek out proper BACE1 inhibition strategies that avoid the goal of maximal and extensive Aβ level lowering in AD brains.

To seek out viable modalities to inhibit BACE1, we focus on the feasibility of nucleic acid drugs (NADs), including microRNAs (miRNAs), which have emerged as potential therapeutic targets for healthy aging and most diseases [23,24,25,26,27]. Although therapeutic nucleic acids (TNAs) have made significant progress and achieved clinical successes in recent years, the broader clinical translation remains constrained by brain delivery for crossing the blood–brain barrier (BBB) in AD [28,29,30]. Of note, novel strategies have been developed to promote nucleic acid-based theranostics to penetrate the BBB for tackling AD, including receptor-mediated endocytosis or nanoparticle conjugation strategies, cell-penetrating-based delivery systems, intracerebroventricular infusion, polymers as biomaterial platforms, and exosomes as nanocarriers [28,31,32].

Recently, we have reviewed the potential roles of miRNAs in AD-related pathologies, early diagnostics, and therapeutic strategies [33]. The underlying physiological and pathological implications of these master regulators remain largely unexplored, and miRNA-based treatment options are still in their infancy [34]. Interestingly, brain-enriched miRNAs could exert highly efficient effects on neuronal reprogramming to generate sporadic late-onset AD (LOAD) neurons in a three-dimensional environment, recapitulating pathological features, including Aβ deposition, tauopathy, and neurodegeneration [35]. The advancement of miRNA-based direct reprogramming suggests vital functions of miRNAs in key biological processes in AD pathogenesis. A number of studies have revealed that miR-143-3p is an AD-related miRNA exhibiting promising diagnostic potential [36,37,38,39,40,41]. However, the biological functions and molecular mechanisms of miR-143-3p in AD brains remain largely unknown.

Histopathologically, the deposition of senile plaques composed of Aβ primarily in the hippocampus and cortex is one of the major hallmarks of AD [42]. To recapitulate major pathological characteristics of AD, 5×FAD mice harboring five familial AD mutations have been created [43] and employed worldwide. Since BACE1 exerts an early role in AD pathogenesis, its elevation in transgenic mouse brains has gained great interest [44]. Surprisingly, even in wild-type mice, higher levels of BACE1 were found in certain brain regions, such as the mossy fiber pathway, instead of the whole hippocampus [44]. Notably, whether the regional location of BACE1 affects the therapeutic effect in AD remains elusive.

In this study, we found that miR-143-3p was inversely correlated with BACE1 levels in AD brains and directly inhibited BACE1 expression. We further explored the expression signature of BACE1 in the hippocampus and cortex of 5×FAD mouse models of AD at different ages. Surprisingly, in the cornu ammonis 1 (CA1) subfield, instead of the whole hippocampus, BACE1 levels were observed to be increased with age. To exploit the potential of regional modulation of BACE1, we took advantage of adeno-associated virus (AAV)-based gene therapy, which has emerged as the preferred choice in clinical applications [45], and restored miR-143-3p levels in the CA1 of the hippocampus. Notably, suppression of BACE1 via miR-143-3p restoration in the CA1 subfield reduced Aβ plaques and ameliorated cognitive deficits in AD mice.

## 2. Materials and Methods

### 2.1. Materials, Cell Lines, and Animals

All chemicals, if not specified, were purchased from Sigma-Aldrich (St. Louis, MO, USA) and employed without purification, including dimethyl sulfoxide (DMSO), ammonium persulfate (APS), and N,N,N’,N’-tetramethylethylenediamine (TMEDA). The protease and phosphatase inhibitor cocktail was purchased from TargetMol (Shanghai, China). RNA fluorescence in situ hybridization (FISH) kit and biotinylated probes were purchased from GenePharma (Shanghai, China). BACE1 (5606) and Aβ (8243) antibodies were purchased from Cell Signaling Technology (Boston, MA, USA). The human neuroblastoma SH-SY5Y cells, human embryonic kidney 293 (HEK293) cells, and mouse neuroblastoma N2a cells were obtained from the Stem Cell Bank/Stem Cell Core Facility (Shanghai, China). SH-SY5Y cells were cultured in Dulbecco’s modified Eagle’s medium/nutrient mixture F-12 (DMEM/F12) (Gibco, New York, NY, USA) supplemented with 10% (*v*/*v*) fetal bovine serum (FBS) (Gibco). HEK293 and N2a cells were cultured in high-glucose DMEM (Gibco) containing 10% FBS. Cultured cells were incubated at 37 °C with 95% air and 5% CO_2_. The male 5×FAD mice were purchased from the Jackson Laboratory (strain number: #034848-JAX). Mice were randomized to different groups, with 8 mice in each group. All the mice were marked with ear tags. Analyses were conducted with operators blinded to the treatment groups. All mouse studies were carried out following the protocols approved by the Institutional Animal Care and Use Committee of Fujian Medical University (approval number: IACUC FJMU 2024-0010).

### 2.2. Cell Transfection

Cell transfection was performed using TurboFect transfection reagent (Thermo Fisher Scientific, Rockford, IL, USA). The miR-143-3p mimics and the corresponding control (negative control, NC) were purchased from GenePharma (Shanghai, China).

### 2.3. RNA Isolation and Quantitative Real-Time PCR (qRT–PCR)

Total RNA was extracted with the NucleoZOL reagent (Macherey-Nagel, Düren, Germany). Reverse transcription was then carried out using a Transcriptor First Strand cDNA Synthesis Kit (Roche, Indianapolis, IN, USA). MiRNAs were extracted using a miRNA Extraction Kit (HaiGene, Harbin, Heilongjiang, China), and cDNA was synthesized using a One Step miRNA cDNA Synthesis Kit (HaiGene). qRT–PCR was performed using FastStart Universal SYBR Green Master Mix (Roche) in a QuantStudio Real-Time PCR system (Applied Biosystems, Waltham, MA, USA). For amplification of human BACE1, the primers h-BACE1-F (5′-TGCCATCACTGAATCAGACAAGT-3′) and h-BACE1-R (5′-AGTCAAAGAAAGGCTCCAGGG-3′) were employed. For amplification of human β-actin, the primers h-β-actin-F (5′-AGGATTCCTATGTGGGCGAC-3′) and h-β-actin-R (5′-ATAGCACAGCCTGGATAGCAA-3′) were employed. For amplification of mouse BACE1, the primers m-BACE1-F (5′-GACCACTCGCTATACACGGG-3′) and m-BACE1-R (5′-TTCTCCGTCTCCTTGCAGTC-3′) were employed. For amplification of mouse β-actin, the primers m-β-actin-F (5′-CGATATCGCTGCGCTGGTC-3′) and m-β-actin-R (5′-AGGTGTGGTGCCAGATCTTC-3′) were employed. For miR-143-3p amplification, the primers miR-143-3p-F (5′-CAGTGAGATGAAGCACTGTAG-3′) and miR-143-3p-R (5′-GGTCCAGTTTTTTTTTTTTTTTGAG-3′) were used. For U6 small nuclear RNA amplification, the primers U6-F (5′-CTCGCTTCGGCAGCACA-3′) and U6-R (5′-AACGCTTCACGAATTTGCGT-3′) were used. Expression was determined by the 2-ΔΔCt method and normalized to the β-actin or U6 level.

### 2.4. Western Blot Analysis

Western blot analysis was carried out as described previously [46,47,48]. Briefly, cells and tissue samples were lysed by RIPA buffer (150 mM NaCl, 0.5% EDTA, 50 mM Tris, and 0.5% NP40). Protein concentration was measured using the BCA Protein Assay Kit (Beyotime Biotechnology, Shanghai, China). Protein lysates were separated by SDS-PAGE, transferred onto PVDF membranes, blocked with 5% non-fat milk for 2 h at room temperature, incubated with the indicated antibodies at 4 °C overnight, incubated with horseradish peroxidase-conjugated secondary antibodies for 1 h at room temperature, and visualized with an ECL chemiluminescence system.

### 2.5. Immunofluorescence Staining

Samples were fixed with 4% paraformaldehyde for at least 24 h, dehydrated via a gradient sucrose solution, frozen in optimal cutting temperature (OCT) medium, cut using a cryotome, mounted on slides, incubated with the indicated antibodies at 4 °C overnight, washed in TBST three times, incubated with secondary antibodies for 1 h at room temperature, and imaged using a fluorescence microscope.

### 2.6. Plasmids

The 3’UTR of human BACE1 containing the miR-143-3p binding sites was inserted into the pmirGLO Dual-Luciferase miRNA Target Expression Vector (Promega, Madison, WI, USA) digested with Xho I and Sal I to construct the human BACE1 wild-type reporter vector (h-BACE1 3’UTR WT). The binding sites were mutated (TCATCTC to AGTAGAG) to construct the mutant reporter vector (h-BACE1 3’UTR MUT). The 3’UTR of mouse BACE1 containing the miR-143-3p binding sites was inserted into the pmirGLO Dual-Luciferase miRNA Target Expression Vector (Promega) digested with Nhe I and Sal I to construct the mouse BACE1 wild-type reporter vector (m-BACE1 3’UTR WT). The binding sites were mutated (TCATCTC to GACGAGA) to construct the mutant reporter vector (m-BACE1 3’UTR MUT).

### 2.7. Luciferase Reporter Assay

HEK293 or N2a cells were cotransfected with miR-143-3p or NC and the BACE1 3’UTR WT or BACE1 3’UTR MUT reporter vector. Forty-eight hours after transfection, cells were harvested for measurement of luciferase activity using the Dual-Luciferase Reporter Assay System (Promega, Madison, WI, USA).

### 2.8. Solid-Phase Sandwich ELISA of Secreted Aβ40 and Aβ42

The amount of secreted Aβ was assessed with a Human/Rat Beta Amyloid [40] ELISA Kit and a Human/Rat Beta Amyloid [42] ELISA Kit (FUJIFILM Wako Pure Chemical Corporation, Osaka, Japan) as previously described [49]. The data are shown as percentages compared with control culture values.

### 2.9. Construction and Production of AAV

The AAV was prepared using triple-plasmid transfection of HEK293 cells. The main AAV plasmid contained AAV2 ITRs. Separate Rep/Cap plasmid and the helper plasmid provided components of the viral replication machinery and the capsid proteins of selected AAV serotypes. After HEK293 cell lysis, viral particles were purified by CsCl gradient ultracentrifugation. And AAVs were titered by quantitative polymerase chain reaction. The viral tools were all packaged by BrainVTA (BrainVTA Co., Ltd., Wuhan, China).

### 2.10. Stereotaxic Brain Injection of AAV

After anesthesia through isoflurane inhalation, mice were placed in a stereotactic frame. A small burr hole was drilled via the skull at the following coordinates: AP: ±1.0 mm, ML: −1.95 mm, and DV: −1.5 mm relative to the bregma. The AAV-miR-143-3p viral vectors (catalog# PT-6615) and controls (catalog# PT-1316) were constructed and supplied by BrainVTA [50,51]. Stereotaxic injection of AAV-miR-143-3p or controls was performed.

### 2.11. Behavioral Tests

Behavioral tests were performed by operators blinded to the genotype and treatment of mice. All the tests were carried out during the light period. The behavioral tests, including the open field test, the novel object recognition test, the Morris water maze test, the Y maze test, and the nesting test, were performed as described in our previous study [52]. Briefly, for the open field test, all mice were allowed for exploration of open area (40 cm × 40 cm arena) for five minutes. The arena was sterilized with 75% alcohol for every test. The distance traveled and time spent in the indicated region for all mice were analyzed by Smart software (version 3.0.06) from Panlab Harvard Apparatus (Barcelona, Spain). For the novel object recognition test, all mice were allowed to explore the arena freely for ten minutes for habituation. Identical object A and object B were placed inside the arena, and every tested mouse was located at the same starting place and allowed to explore them freely for five minutes. 24 h later, object C was used to replace object A, and every mouse was placed in the arena for exploration of objects for five minutes. The time spent and recognition index for every object for all mice were analyzed by Smart software. For the Morris water maze test, every mouse was trained with a visible platform test (the first day) and a hidden platform test (the second to fifth days), and trials every day were performed with an interval of around twenty minutes. For training, all mice were allowed to stay on the platform for five minutes when they arrived on the platform within one minute. Alternatively, the mice were put on the platform directly for twenty seconds. On the sixth day, a probe test was conducted, and every mouse was analyzed for swimming freely for one minute without the platform. The data for all mice were analyzed by Smart software. For the Y maze test, an apparatus with three identical arms (38 cm × 8 cm × 16.5 cm) was used. For the training section, all mice were put at the end of one arm, while the second arm was blocked as the novel arm, and the third arm was open as the familiar arm. All mice were allowed to explore for five minutes. The apparatus was sterilized with 75% alcohol for every test. For the test section, every mouse was allowed to explore freely for three minutes in three arms, which were all open. The movement of mice, entry numbers, and the time spent in the novel arm for all mice were analyzed by Smart software. For the nesting test, every mouse was kept in a new cage alone for 24 h. Paper strips were put into the cages at 6:00 PM. The construction of the nest was analyzed in a blinded manner using a 5-point Deacon scale.

### 2.12. Brain Samples

Brain hippocampal tissues were collected from individuals with AD and age-matched control subjects (Tables S1 and S2). The study on human samples was reviewed and approved by the Ethics Committee of Fujian Medical University (2024-12). In most cases, the brain tissue samples used in this research were harvested within 30 h postmortem.

### 2.13. Statistical Analysis

Statistical analyses were performed using GraphPad Prism software (version 8.3.0, GraphPad, San Diego, CA, USA). Data are expressed as the mean ± standard deviation (SD) of three independent experiments. Statistical significance was analyzed by either a two-tailed unpaired t test or one-way analysis of variance (ANOVA) followed by Tukey’s post hoc test. *p* < 0.05 was considered significant unless otherwise stated.

## 3. Results

### 3.1. MiR-143-3p Is Inversely Correlated with BACE1 in the Postmortem Hippocampus and Directly Targets BACE1 to Reduce Aβ

**Figure 1:**
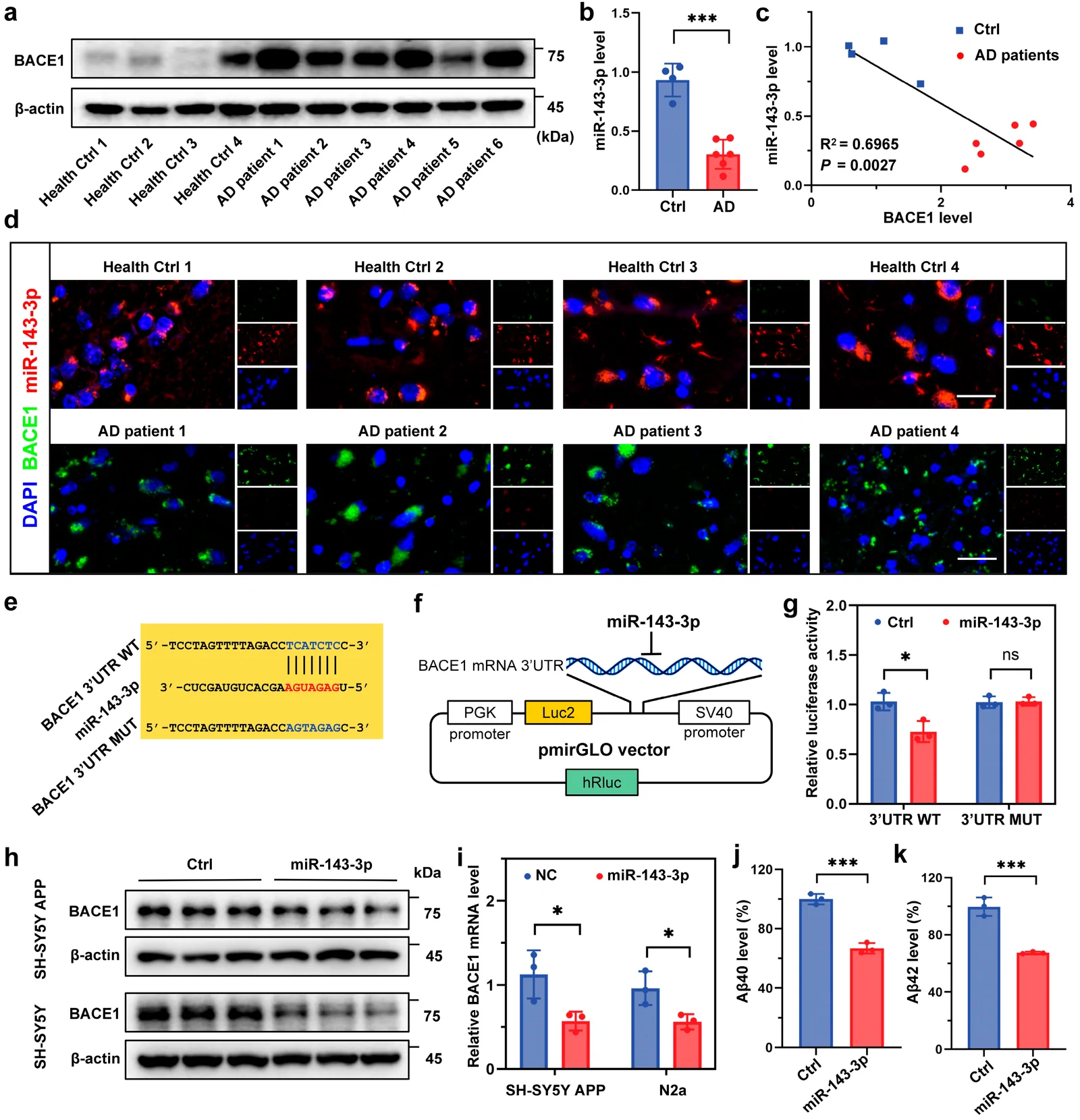
MiR-143-3p levels in the hippocampal tissue samples of AD patients are inversely correlated with BACE1 expression and directly target BACE1 to inhibit Aβ production. (**a**) Western blotting analysis of BACE1 in the postmortem hippocampus of patients with AD and healthy individuals. (**b**) Levels of miR-143-3p quantified by RT-qPCR in the hippocampal tissue samples from AD patients and age-matched controls. (**c**) Linear regression analysis of the correlation between miR-143-3p and BACE1 levels. (**d**) Representative images of miR-143-3p FISH and BACE1 immunofluorescence in AD hippocampus and healthy controls, with nuclei stained with DAPI. Scale bar: 30 μm. (**e**,**f**) The 3’UTR of BACE1 mRNA containing the wild-type (WT) or mutant (MUT) sequence of the potential binding site of miR-143-3p cloned into the pmirGLO dual-luciferase vector. (**g**) The relative luciferase activity in HEK293 cells cotransfected with miR-143-3p mimics or controls and the pmirGLO vector containing the WT or MUT sequence. (**h**) Western blotting analysis of BACE1 in SH-SY5Y APP cells and SH-SY5Y cells treated with miR-143-3p or controls. (**i**) RT-qPCR analysis of BACE1 mRNA in SH-SY5Y APP cells and N2a cells treated with miR-143-3p or controls. (**j**,**k**) Levels of Aβ40 and Aβ42 in cell culture supernatants after treatment with miR-143-3p or controls detected by a solid-phase sandwich ELISA assay. Data are presented as the mean ± SD (* *p* < 0.05; *** *p* < 0.001; ns, not significant). The original Western blot images can be found in the Supplementary Materials File S1.

Owing to the unique rate-limiting role in the amyloidogenic pathway, BACE1 has aroused great expectations for AD therapeutics, but human clinical trials involving most BACE1 inhibitors have been halted [12,16,18,53]. To explore more viable strategies targeting BACE1, we focus on emerging modalities, including nucleic acid-based therapeutics [23,25,26]. In our previous work, we observed that miR-143-3p is decreased in the hippocampal tissues of AD individuals compared with age-matched controls [54], but the biological functions and molecular mechanisms of miR-143-3p in AD brains remain largely unknown. Interestingly, upregulated BACE1 levels and downregulated miR-143-3p levels in the hippocampus of AD patients were found to be inversely correlated (R^2^ = 0.6965) (Figure 1a–c and Figure S1). Furthermore, through a combination of miRNA fluorescence in situ hybridization (FISH) and protein immunofluorescence [55] in human brain hippocampal tissues, we observed that high levels of BACE1 and low levels of miR-143-3p were expressed in AD patients compared with controls (Figure 1d). These findings suggest that miR-143-3p levels might be inversely correlated with BACE1 expression in AD brains.

Given that miRNAs serve as crucial gene expression regulatory factors at the post-transcriptional level [24], we further investigated whether miR-143-3p could directly affect BACE1 protein expression. Using the TargetScan database to analyze the seed sequence of miR-143-3p and the 3’UTR sequences of BACE1 mRNA, we found that miR-143-3p possessed the capacity to bind to BACE1 mRNA. To validate their direct interaction, we cloned the 3’UTR of BACE1 mRNA containing the wild-type (WT) or mutant (MUT) sequence of the potential binding site into the pmirGLO dual-luciferase vector (Figure 1e,f). The dual-luciferase reporter assay indicated that cotransfection of miR-143-3p with the vector containing the WT binding sequence inhibited the activity of the firefly luciferase reporter, whereas the luciferase activity was restored when the putative binding site was mutated, demonstrating that BACE1 was a direct target of miR-143-3p (Figure 1g and Figurs S2). To better assess the effects of miR-143-3p on BACE1 protein expression and Aβ production, we took advantage of SH-SY5Y cells stably transfected with APP (SH-SY5Y APP) as an AD cell model. Compared with the corresponding control, treatment with miR-143-3p mimics could suppress the protein expression of BACE1 (Figure 1h and Figure S3). The BACE1 mRNA levels were also downregulated by miR-143-3p (Figure 1i), indicating that miR-143-3p could induce degradation of the BACE1 mRNA. Furthermore, the levels of total Aβ40 and Aβ42 secretion were also decreased after miR-143-3p treatment in SH-SY5Y APP cells (Figure 1j,k). The data show that miR-143-3p can directly target BACE1 and reduce its levels, leading to decreased Aβ generation, suggesting a potential therapeutic strategy based on the intrinsic mechanism.

### 3.2. BACE1 Levels Are Elevated with Age in the CA1 Subfield of the Hippocampus in AD Model Mice

**Figure 2:**
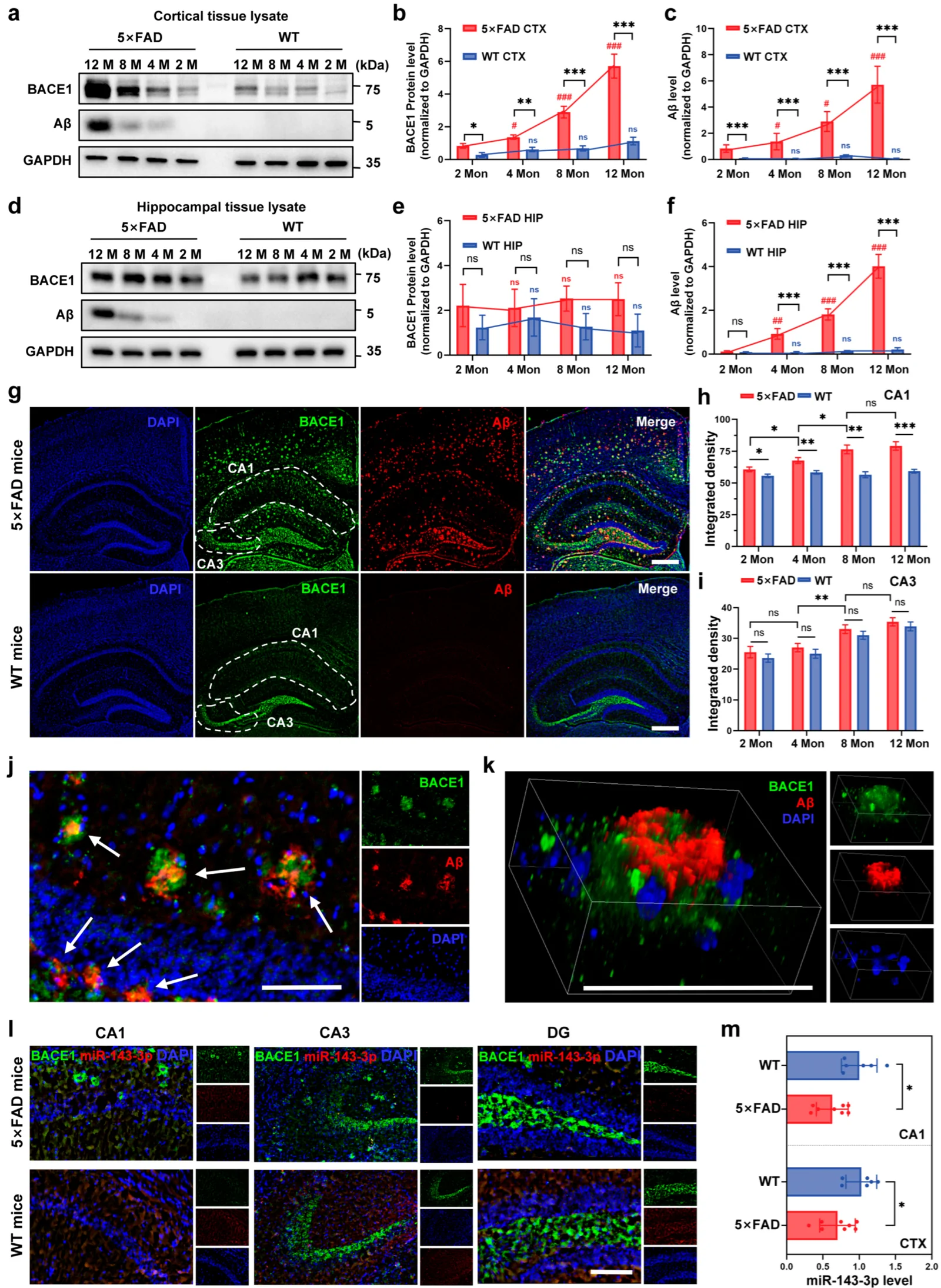
BACE1 levels are regionally upregulated in the hippocampal CA1 area of 5×FAD mice with age. (**a**–**c**) Western blotting analysis and quantification of BACE1 and Aβ levels in the cortex of AD model mice and WT mice at 2, 4, 8, and 12 months of age. (**d**–**f**) Western blotting analysis and quantification of BACE1 and Aβ levels in the hippocampus of AD model mice and WT mice at 2, 4, 8, and 12 months of age. (**g**) Representative immunostaining images of BACE1 and Aβ in the CA1 and CA3 subfields of the hippocampus in AD and WT mice, with nuclei stained with DAPI. Scale bar: 500 μm. (**h**) Quantification of BACE1 levels in the hippocampal CA1 area of AD mice and WT mice at 2, 4, 8, and 12 months of age. (**i**) Quantification of BACE1 levels in the hippocampal CA3 area of AD mice and WT mice at 2, 4, 8, and 12 months of age. (**j**) Representative immunostaining images of BACE1 and Aβ in the brains of AD mice, with nuclei stained with DAPI. Scale bar: 100 μm. (**k**) Representative three-dimensional simulation images of BACE1 and Aβ in the brains of AD mice, with nuclei stained with DAPI. Scale bar: 200 μm. (**l**) Representative images of miR-143-3p FISH and BACE1 immunofluorescence in the hippocampal CA1, CA3, and DG areas of AD mice and WT mice, with nuclei stained with DAPI. Scale bar: 200 μm. (**m**) Levels of miR-143-3p quantified by RT-qPCR in the hippocampal CA1 area and cortex of AD mice and WT mice. Data are presented as the mean ± SD (* *p* < 0.05; ** *p* < 0.01; *** *p* < 0.001; # *p* < 0.05; ## *p* < 0.01; ### *p* < 0.001; ns, not significant). The original Western blot images can be found in the Supplementary Materials File S1.

To fully exploit the therapeutic potential of miR-143-3p on BACE1 suppression in AD brains, we first examined the spatio-temporal expression of BACE1 in the hippocampus and cortex of 5×FAD model mice at 2, 4, 8, and 12 months of age via Western blotting analyses. As expected, in the cortical tissues of AD mice, both BACE1 and Aβ levels were significantly elevated with age compared with control groups (Figure 2a–c). However, to our surprise, in the hippocampal tissues, although Aβ generation was increased with age in 5×FAD mice, the alteration of BACE1 expression was not statistically significantly associated with age (Figure 2d–f).

To further seek out whether BACE1 levels are regionally different within the hippocampus, we analyzed the expression profiles of BACE1 and Aβ in mouse brain tissue sections through immunofluorescence microscopy. Compared with WT mice, the formation of Aβ and upregulation of BACE1 were obvious in the hippocampus and cortex of 5×FAD mice (Figures S4 and S5). BACE1 levels were visualized in the brains and compared in the hippocampal areas CA1 and CA3, which are important for learning and memory [56]. Of note, in the CA1 subfield of the hippocampus, the expression of BACE1 was increased in AD mice with age when compared with WT mice (Figure 2g,h). By contrast, in the CA3 subfield of the hippocampus, the rise in BACE1 with age was not significant (Figure 2i). The spatial co-localization of BACE1 and Aβ was observed at the site of amyloid plaques in the brains of AD model mice (Figure 2j). Three-dimensional simulation technology further indicated that BACE1 was located around Aβ plaques (Figure 2k).

Through the combination of miRNA FISH and protein immunofluorescence in mouse brain hippocampal tissues, high levels of BACE1 and low levels of miR-143-3p were observed in the hippocampal CA1 area compared with controls (Figure 2l). By contrast, the background levels of BACE1 in other hippocampal subregions, such as CA3 and dentate gyrus (DG), were relatively high even in WT mice, which might contribute to insignificant alterations in BACE1 levels in the hippocampus of AD mice of different ages. Moreover, compared with WT controls, the levels of miR-143-3p were decreased in the CA1 subfield of the hippocampus and cortex in AD mice (Figure 2m). The above data suggest that the expression of BACE1 might be regionally upregulated in the CA1 area of the hippocampus in 5×FAD model mice with age compared to WT control mice.

### 3.3. AAV-Mediated MiR-143-3p Restoration in the CA1 Subfield of the Hippocampus Improves the Cognitive Function of AD Model Mice

**Figure 3:**
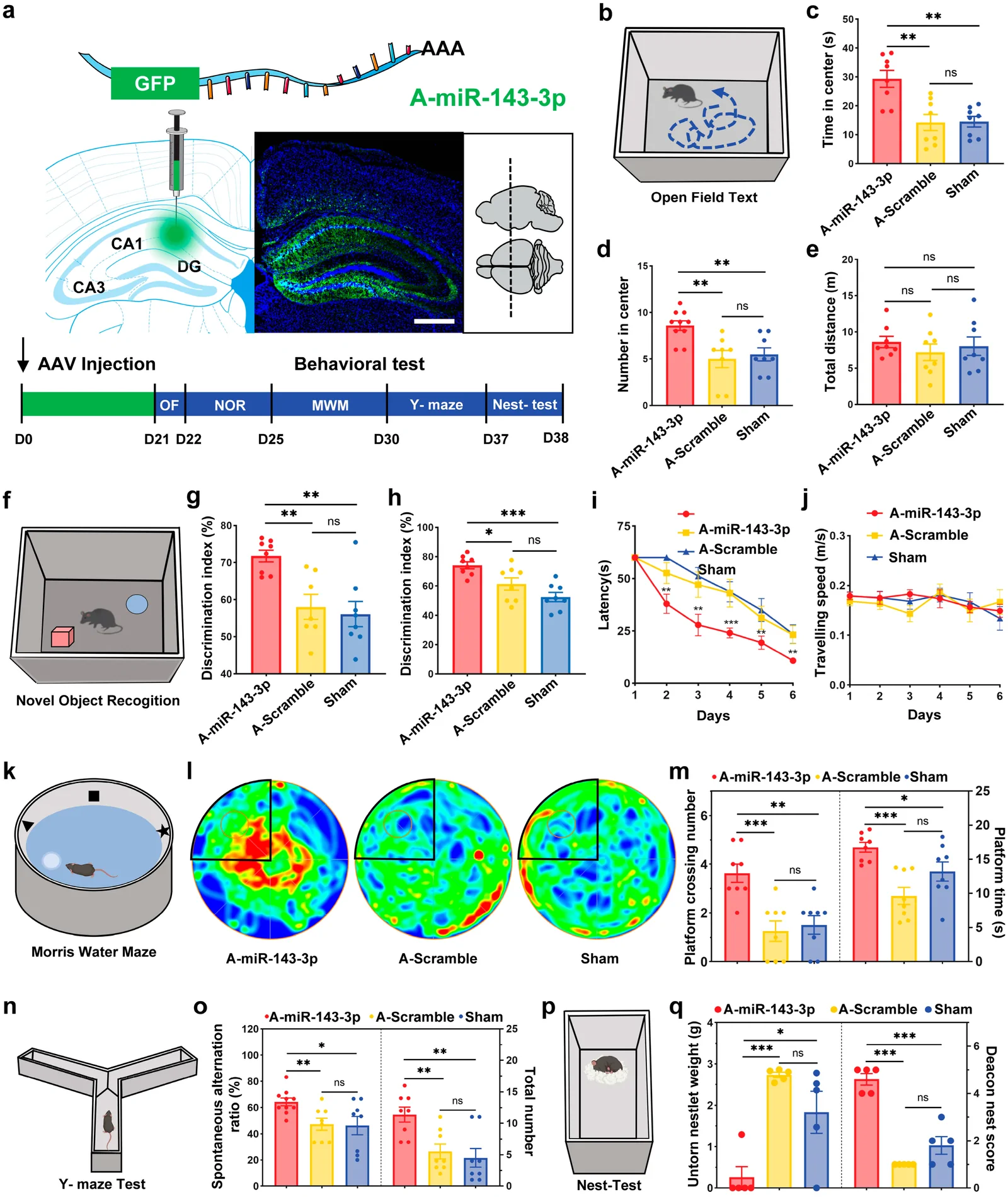
AAV-mediated restoration of miR-143-3p in the hippocampal CA1 area through stereotaxical injection rescues the cognitive performance in 5×FAD mice. (**a**) Representative images depicting the site of stereotaxic injection and the expression of AAV in the hippocampus of AD mice, as well as a schematic timeline illustrating AAV treatment and behavioral tests. Scale bar: 500 μm. (**b**–**e**) Time spent in the center zone, frequency in the center zone, and total moving distance for AD mice of three groups in the OF test. (**f**–**j**) Discrimination index of the time, the number, and the travelling speed of AD mice in three groups in the NOR test. (**k**–**m**) Representative heatmaps of the probe trial, target crossings, and exploring time in the targeted zone after removing the platform for AD mice of three groups in the MWM test. (**n**,**o**) Percentage of alterations and number of arm entries for AD mice in three groups in the Y maze test. (**p**,**q**) Untorn nestlet weight and deacon nest score in AD mice of three groups in the nesting test. Data are presented as the mean ± SD (* *p* < 0.05; ** *p* < 0.01; *** *p* < 0.001; ns, not significant).

For most BACE1 inhibitors, the clinical trials were discontinued as a result of ineffective cognitive improvement or even cognitive worsening [12,16,18]. For the cellular basis underlying spatial learning and memory, hippocampal CA1 long-term potentiation (LTP) has been found to play a pivotal role [57]. Recent findings indicated that CA1 neurons were significantly reduced, and aggravated Aβ plaques were observed in the CA1 subregion [58]. Thus, we next investigated whether restoring miR-143-3p in the hippocampal CA1 area could affect the cognitive ability of 5×FAD mice by AAV injection, which has promising applications in clinical gene therapy [45]. We employed an AAV expressing miR-143-3p or an empty vector and stereotaxically injected them into the CA1 subfield of the hippocampus in 6-month-old 5×FAD mice [59,60,61,62], together with a sham operation group (Figure 3a, Figures S6 and S7). The regional distribution of AAV-miR-143-3p expression was mainly in the CA1 subfield of the hippocampus (Figures S8 and S9). Three weeks later, the open field (OF) test, the novel object recognition (NOR) test, the Morris water maze (MWM) test, the Y maze test, and the nesting test were sequentially executed to assess the behavioral and cognitive performance of the mice.

In the OF test, AAV-miR-143-3p-treated AD mice spent more amounts of time with more frequency in the open space compared with other control groups, while the total moving distance showed no significant difference among the three groups (Figure 3b–e and Figure S10). In the NOR test, AD mice treated with AAV-miR-143-3p exhibited elevated interest in questing novel objects, determined by increased discrimination index of time and number and decreased latency time, while the travelling speed was similar among the three groups (Figure 3f–j). In the MWM test, AAV-miR-143-3p treatment enhanced platform crossing numbers and swimming time in the target quadrant of AD mice compared to the other two groups (Figure 3k–m). In the Y maze test, 5×FAD mice treated with AAV-miR-143-3p showed increased spontaneous alternation and total number of arm entries, while the three groups presented indistinct total distance (Figure 3n,o and Figure S11). In the nesting test, the treatment with AAV-miR-143-3p decreased untorn nestlet weight and increased deacon nest scores in AD mice compared with control mice (Figure 3p,q and Figure S12). These results suggest that restoring miR-143-3p in the hippocampal CA1 area via stereotaxic injection of AAV can ameliorate cognitive impairment in 5×FAD model mice.

### 3.4. AAV-Mediated MiR-143-3p Restoration in the CA1 Subfield of the Hippocampus Reduces BACE1 and Aβ Levels in AD Model Mice

**Figure 4:**
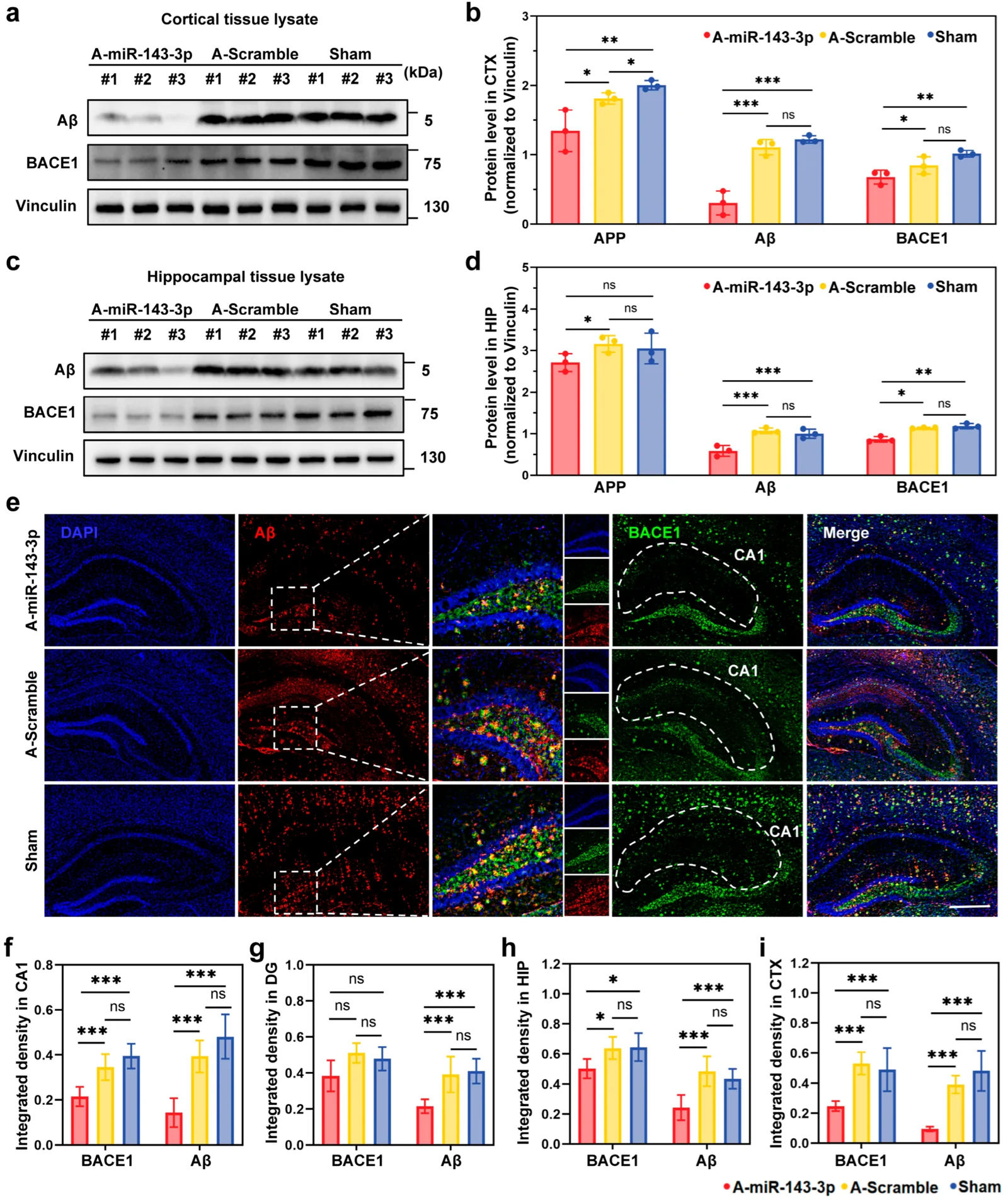
AAV-mediated restoration of miR-143-3p in the hippocampal CA1 area through stereotaxic injection attenuates BACE1 and Aβ levels in 5×FAD mice. (**a**,**b**) Western blotting analysis and quantification of BACE1 and Aβ levels in the cortex of AD model mice in three groups. (**c**,**d**) Western blotting analysis and quantification of BACE1 and Aβ levels in the hippocampus of AD model mice in three groups. (**e**) Representative immunostaining images of Aβ and BACE1 in the brains of AD mice, with nuclei stained with DAPI. Scale bar: 500 μm. (**f**) Quantification of Aβ and BACE1 levels in the CA1 area of AD mice. (**g**) Quantification of Aβ and BACE1 levels in the DG area of AD mice. (**h**) Quantification of Aβ and BACE1 levels in the hippocampus of AD mice. (**i**) Quantification of Aβ and BACE1 levels in the cortex of AD mice. Data are presented as the mean ± SD (* *p* < 0.05; ** *p* < 0.01; *** *p* < 0.001; ns, not significant). The original Western blot images can be found in the Supplementary Materials File S1.

To further investigate whether the miR-143-3p-induced cognitive improvement was associated with BACE1 expression and Aβ generation in the brains, we detected their levels in the cortex and hippocampus of 5×FAD model mice in three groups. The results indicated that in both cortical and hippocampal tissues, AAV-miR-143-3p treatment significantly downregulated the levels of BACE1 and Aβ (Figure 4a–d). Moreover, we examined the spatial expression of BACE1 and Aβ in the brains via immunofluorescence microscopy. Immunostaining revealed that both BACE1 expression and Aβ levels were reduced in the CA1 subfield of the hippocampus when compared with other groups (Figure 4e,f). Nevertheless, the differences in BACE1 levels in the DG subregions were not significant (Figure 4g), which might be explained by the existence of high background levels. When the whole hippocampus was calculated, BACE1 levels were found to be downregulated after miR-143-3p treatment, although the differences became smaller compared with the CA1 area (Figure 4h). Furthermore, in the cortex of AD mice, treatment with miR-143-3p also resulted in a reduction in BACE1 (Figure 4i). In addition, in the primary cultured neurons of 5×FAD model mice, miR-143-3p treatment decreased BACE1 expression levels (Figures S13 and S14). These data suggest that restoring miR-143-3p levels in the hippocampal CA1 area can decrease the expression of BACE1 and Aβ in the brains of 5×FAD model mice.

### 3.5. AAV-Mediated MiR-143-3p Restoration in the CA1 Subfield of the Hippocampus Modulates Microglial Polarization in AD Model Mice

**Figure 5:**
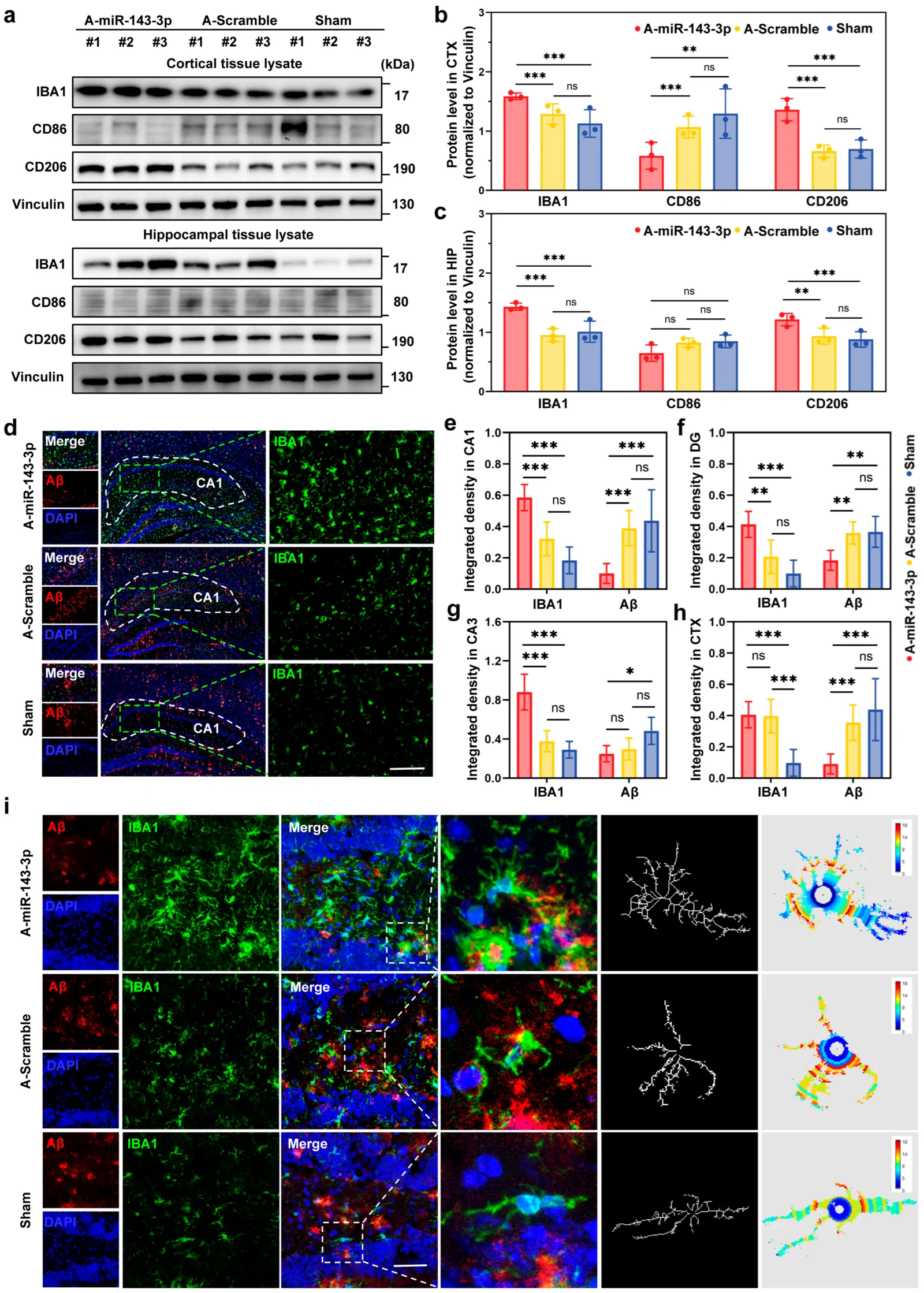
AAV-mediated restoration of miR-143-3p in the hippocampal CA1 area through stereotaxic injection promotes the microglia polarization toward the anti-inflammatory phenotype in 5×FAD mice. (**a**) Western blotting analysis of IBA1, CD86, and CD206 levels in the cortex and hippocampus of AD model mice in three groups. (**b**,**c**) Quantification of IBA1, CD86, and CD206 levels in the cortex and hippocampus of AD model mice in three groups. (**d**) Representative immunostaining images of IBA1 and Aβ in the brains of AD mice in three groups, with nuclei stained with DAPI. Scale bar: 100 μm. (**e**) Quantification of IBA1 and Aβ levels in the CA1 area of AD mice in three groups. (**f**) Quantification of IBA1 and Aβ levels in the DG area of AD mice in three groups. (**g**) Quantification of IBA1 and Aβ levels in the CA3 area of AD mice in three groups. (**h**) Quantification of IBA1 and Aβ levels in the cortex of AD mice in three groups. (**i**) Representative high-resolution images of IBA1 and Aβ in the brains of AD mice in three groups, with nuclei stained with DAPI. Scale bar: 50 μm. Data are presented as the mean ± SD (* *p* < 0.05; ** *p* < 0.01; *** *p* < 0.001; ns, not significant). The original Western blot images can be found in the Supplementary Materials File S1.

Recent findings indicated that deletion of BACE1 in microglia could reduce amyloid plaques and improve cognitive performance [63], thus we further explored whether restoring miR-143-3p levels affected the polarization of microglia in the hippocampus and cortex of the brain by analyzing microglial markers. Western blotting analysis indicated that the levels of ionized calcium-binding adapter molecule 1 (IBA1) and the anti-inflammatory marker CD206 were increased in both cortical and hippocampal tissues after miR-143-3p treatment (Figure 5a–c), indicating an increase in phagocytic microglia. Immunostaining also showed that IBA1 levels were increased and Aβ levels were decreased in the brains of AD mice after miR-143-3p treatment (Figure 5d–h). Three-dimensional images from a high-resolution microscope further revealed that microglia marked with IBA1 were located around Aβ plaques (Figure 5i and Figure S15), which was consistent with a previous report demonstrating that more microglia in close contact with amyloid plaques could be induced after Bace1 deletion [63]. These results suggest that AAV-mediated miR-143-3p restoration in the brains of AD mice can regulate microglia and adjust the polarization toward an anti-inflammatory phenotype surrounding Aβ plaques.

### 3.6. AAV-Mediated MiR-143-3p Restoration in the CA1 Subfield of the Hippocampus Modulates Neural-Related Genes in AD Model Mice

**Figure 6:**
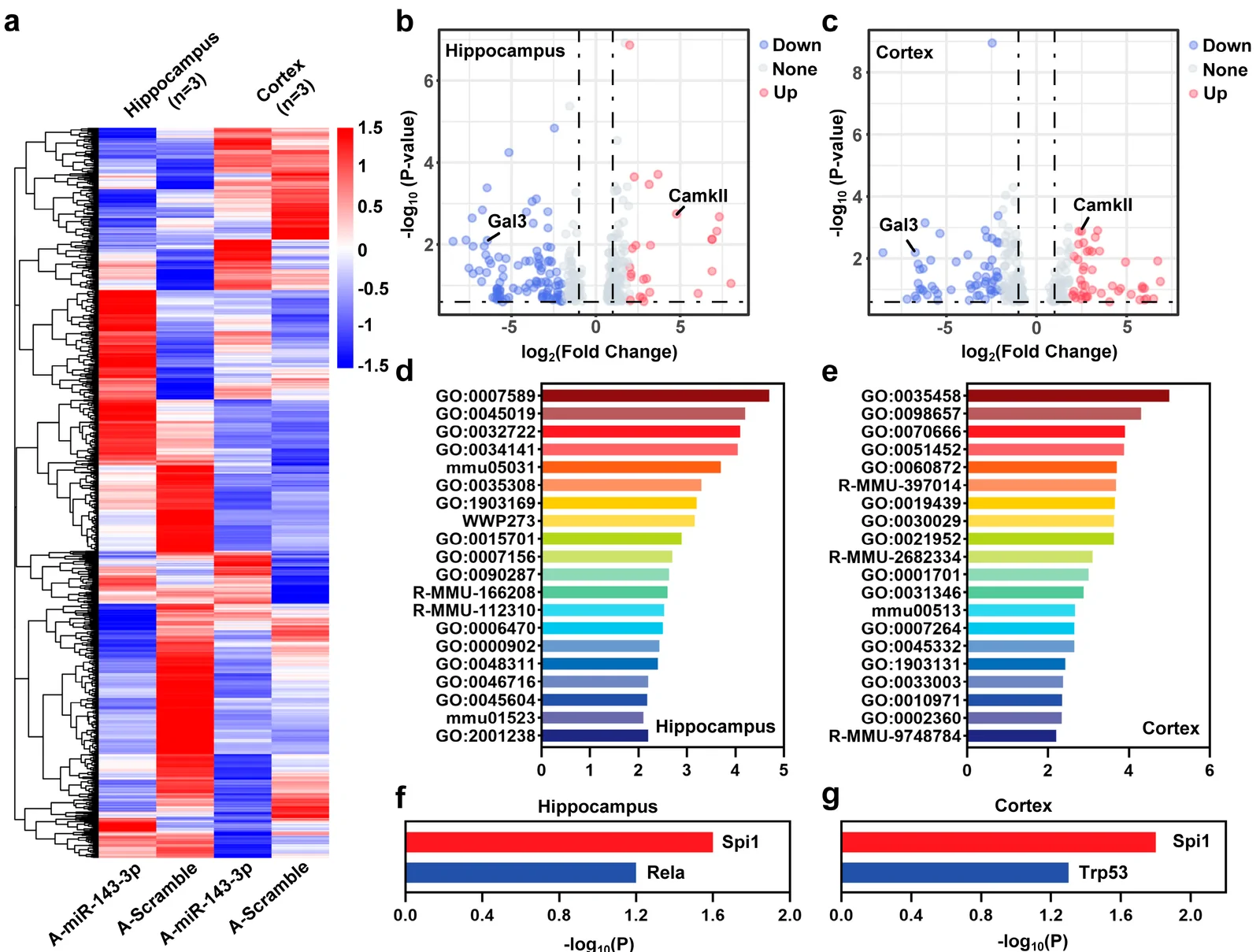
AAV-mediated restoration of miR-143-3p in the hippocampal CA1 area regulates neural-related genes in 5×FAD mice. (**a**) Heatmap of DEGs in the hippocampal or cortical tissues between miR-143-3p-treated groups and control groups. (**b**) The volcano plot showing the distribution of DEGs in the hippocampus between miR-143-3p-treated groups and control groups in the RNA-seq analysis. (**c**) The volcano plot showing the distribution of DEGs in the cortex between miR-143-3p-treated groups and control groups in the RNA-seq analysis. (**d**) GO enrichment analysis of DEGs in the hippocampus. (**e**) GO enrichment analysis of DEGs in the cortex. (**f**,**g**) A shared key regulated gene, Spi1 (PU.1), in both the hippocampus and the cortex.

To further uncover the regulatory genes of miR-143-3p improving the cognitive functions of 5×FAD mice, the transcriptomic profiles in the hippocampal and cortical tissues were characterized to explore neural-related genes (Figure 6a). Differentially expressed genes (DEGs) were identified after filtering the raw data. Interestingly, in both the hippocampus and cortex of miR-143-3p-treated groups, Gal3 (galectin-3) levels were downregulated, while calcium/calmodulin-dependent protein kinase II (CAMKII) levels were upregulated, when compared to the control groups (Figure 6b,c). Gene ontology (GO) enrichment analyses were performed to explore the enriched terms (Figure 6d,e). Notably, further analyses of RNA sequencing results demonstrated a shared key regulated gene, Spi1 (PU.1), in both the hippocampus (Figure 6f) and the cortex (Figure 6g). These results suggest that neural-related genes are regulated in the hippocampus and cortex of AD model mice with miR-143-3p treatment in the CA1 subfield of the hippocampus.

### 3.7. AAV-Mediated MiR-143-3p Restoration in the CA1 Subfield of the Hippocampus Regulates Gal3 and PU.1 in AD Model Mice

**Figure 7:**
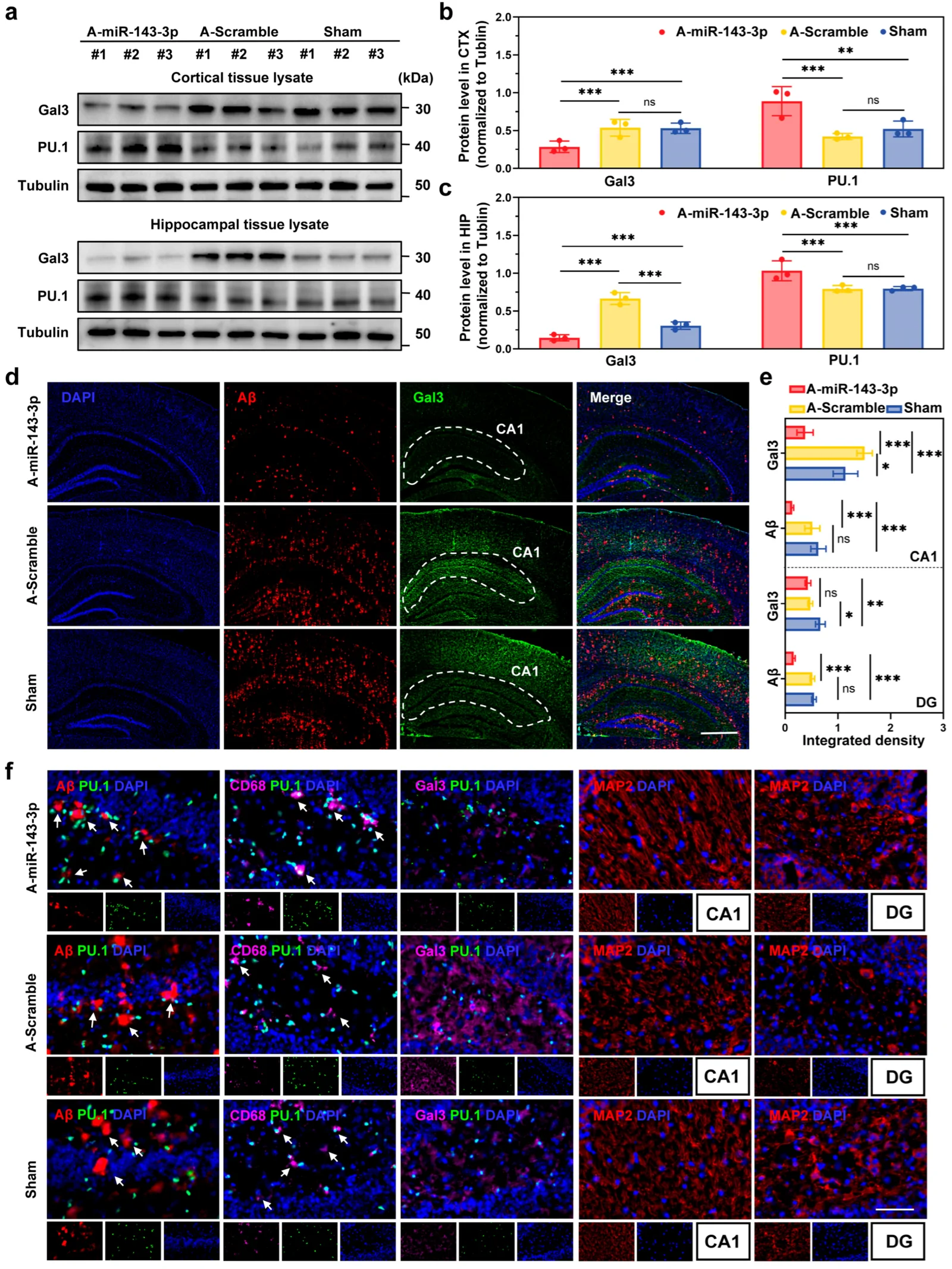
AAV-mediated restoration of miR-143-3p in the hippocampal CA1 area through stereotaxic injection modulates Gal3 and PU.1 levels in 5×FAD mice. (**a**) Western blotting analysis and quantification of Gal3 and PU.1 levels in the cortex and hippocampus of AD model mice in three groups. (**b**) Quantification of Gal3 and PU.1 levels in the cortex of AD model mice in three groups. (**c**) Quantification of Gal3 and PU.1 levels in the hippocampus of AD model mice in three groups. (**d**) Representative immunostaining images of Aβ and Gal3 in the brains of AD mice, with nuclei stained with DAPI. Scale bar: 500 μm. (**e**) Quantification of Aβ and Gal3 levels in the hippocampal CA1 area and DG area of AD mice. (**f**) Representative immunostaining images of Aβ, PU.1, CD68, Gal3, and MAP2 in the brains of AD mice, with nuclei stained with DAPI. Scale bar: 50 μm. Data are presented as the mean ± SD (* *p* < 0.05; ** *p* < 0.01; *** *p* < 0.001; ns, not significant). The original Western blot images can be found in the Supplementary Materials File S1.

Considering that Gal3 [64,65,66] and PU.1 [67,68] have been demonstrated to play critical roles in Aβ pathologies, we next investigated the regulatory effects of miR-143-3p on Gal3 and PU.1 expression in the hippocampus and cortex of the brain. Western blotting analysis revealed that Gal3 levels were downregulated, and PU.1 levels were upregulated in both cortical and hippocampal tissues after miR-143-3p treatment (Figure 7a–c). Immunostaining indicated that both Aβ levels and Gal3 expression were decreased, while PU.1 levels were increased and located around Aβ in the CA1 subfield of the hippocampus compared to the other groups (Figure 7d–f). In addition, CD68 expression around Aβ and MAP2 levels were also induced after treatment with miR-143-3p. These data suggest that Aβ-related Gal3 and PU.1 can be modulated by AAV-mediated miR-143-3p restoration in the brains of AD mice.

### 3.8. AAV-Mediated MiR-143-3p Restoration in the CA1 Subfield of the Hippocampus Attenuates Synaptic Loss in AD Model Mice

**Figure 8:**
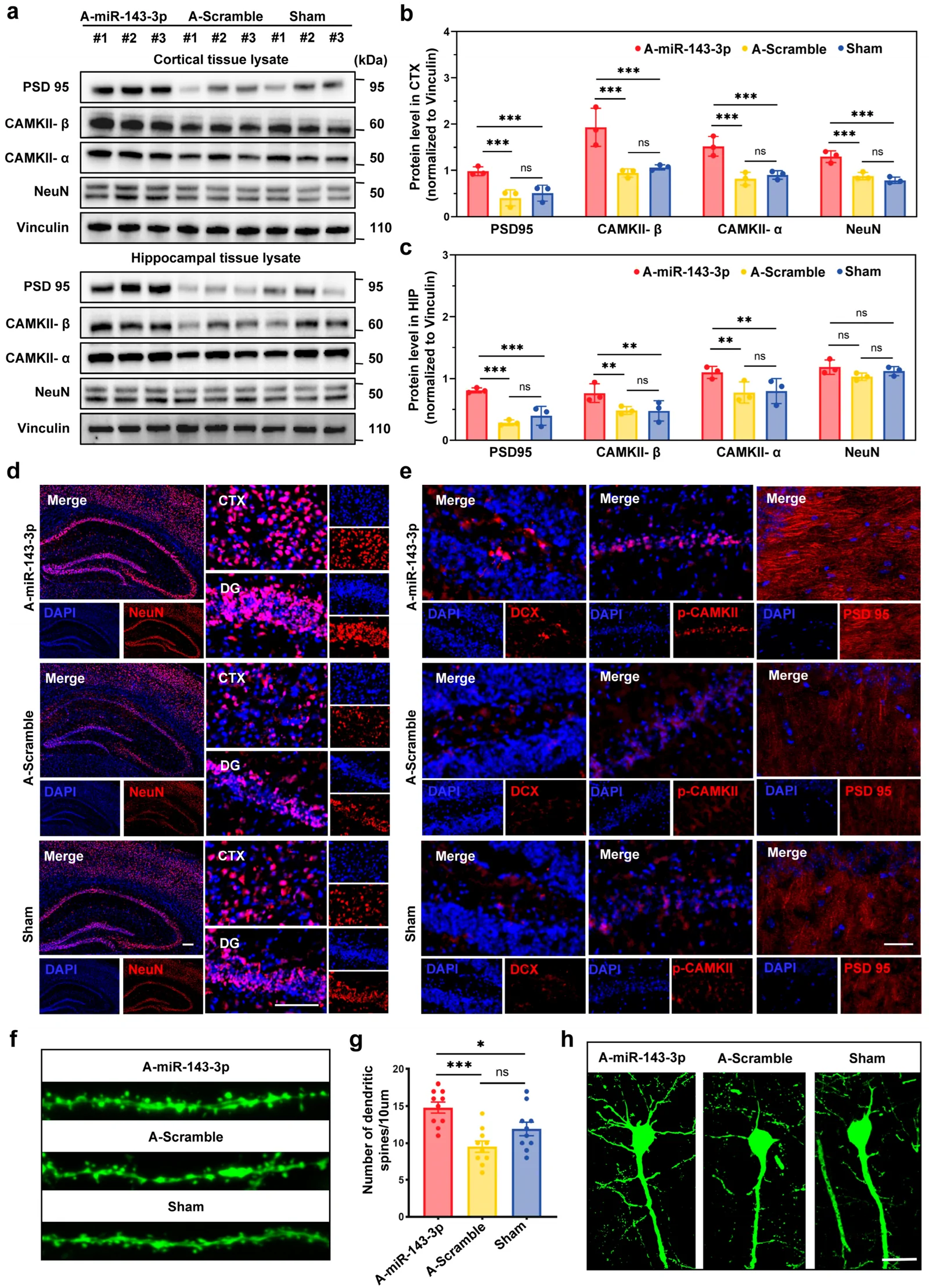
AAV-mediated restoration of miR-143-3p in the hippocampal CA1 area through stereotaxic injection rescues synaptic disorders in 5×FAD mice. (**a**) Western blotting analysis of PSD95, CAMKII, and NeuN levels in the cortex and hippocampus of AD model mice in three groups. (**b**,**c**) Quantification of PSD95, CAMKII, and NeuN levels in the cortex and hippocampus of AD model mice in three groups. (**d**) Representative immunostaining images of NeuN in the brains of AD mice, with nuclei stained with DAPI. Scale bar: 50 μm. (**e**) Representative immunostaining images of DCX, p-CAMKII, and PSD95 in the brains of AD mice, with nuclei stained with DAPI. Scale bar: 50 μm. (**f**) Representative Golgi staining images of dendritic spines in the brains of AD mice in three groups. (**g**) Number of dendritic spines in the brains of AD model mice in three groups. (**h**) Representative Golgi staining images of dendritic trees in the brains of AD mice in three groups. Scale bar: 25 μm. Data are presented as the mean ± SD (* *p* < 0.05; ** *p* < 0.01; *** *p* < 0.001; ns, not significant). The original Western blot images can be found in the Supplementary Materials File S1.

Given that synaptic loss is considered an early event of neurodegenerative diseases, including AD, and correlates with cognitive decline and memory loss [69,70], we further examined the synaptic protective roles of miR-143-3p in the hippocampus and cortex of the brain. Western blotting analysis indicated that the levels of synapse marker protein postsynaptic density 95 (PSD95) and CAMKII were increased in both cortical and hippocampal tissues after treatment with miR-143-3p (Figure 8a–c). The neuronal marker neuronal nuclei (NeuN) was upregulated in cortical tissues, but the difference in hippocampal tissues was not obvious. Immunostaining also showed that NeuN, DCX, p-CAMKII, and PSD95 levels were upregulated in the brains of AD mice (Figure 8d,e). Golgi staining analyses revealed that miR-143-3p treatment restored the density and maturation of dendritic spines (Figure 8f–h).

These data suggest that synaptic disorders can be rescued by AAV-mediated miR-143-3p restoration in the brains of AD mice.

## 4. Discussion

BACE1 regulates the rate-limiting step for Aβ generation [53], but recent BACE1 inhibitors in clinical trials often resulted in various side effects, including cognitive decline [12,16,18]. Here, we demonstrate that miR-143-3p levels are downregulated in the hippocampal tissue samples from AD individuals and are inversely correlated with the expression of BACE1, which is a direct target of miR-143-3p. Our data indicate that in the hippocampus of 5×FAD model mice, BACE1 protein expression is increased with age in the CA1 subfield. We show that through stereotaxical injection of AAV to restore miR-143-3p levels in the hippocampal CA1 area, the cognitive functions of AD model mice can be improved, BACE1 and Aβ levels are downregulated, the polarization of microglia toward the anti-inflammatory phenotype is induced, neural-related genes, including Aβ-related Gal3 and Spi1, are regulated, and synaptic loss is attenuated.

Abnormal Aβ deposition in the brain is hypothesized to be an early hallmark and fundamental cause of both autosomal dominant AD and sporadic LOAD [3,71,72,73]. A recent multicenter study identified that the levels of cerebrospinal fluid (CSF) Aβ42 differed by an estimated 18 years preceding clinical diagnosis of AD [74]. However, currently approved drugs have demonstrated a weak clinical slowing effect rather than halting the progression of cognitive decline [2]. BACE1 functions in the initial step of the amyloidogenic pathway and has long been regarded as a promising target for AD, but clinical trials of various BACE1 inhibitors were discontinued owing to an inability to improve cognitive functions and safety concerns [15,16]. Our findings indicated that BACE1 could be inhibited by miR-143-3p, and the cognitive performance of 5×FAD model mice could be improved by stereotaxic injection of AAV expressing miR-143-3p in the CA1 subfield of the hippocampus. Further analyses showed that BACE1 and Aβ levels were both reduced in the brains of AD mice after treatment with miR-143-3p. Notably, a number of recent studies also highlight the regulatory importance of BACE1 in the brain and the superior therapeutic potential in AD [22,63,75,76,77,78,79]. Among various previously used strategies for BACE1 inhibition, only five candidates entered phase III clinical trials, but all failed to offer cognitive or functional benefits [12,80]. These drugs shared a conserved 2-aminoheterocycle motif to interact with the catalytic aspartates [80]. Notably, the localization of BACE1 in the brain may contribute to the failure of clinical trials, and recent focus on BACE1 therapy has signaled a shift from traditional enzyme inhibitor-based drugs toward novel strategies, including gene therapy [80].

AAV has been regarded as one of the most beneficial gene therapy vectors for brain diseases [80]. A recent study used AAV-delivered single-domain antibodies targeting BACE1 as a novel therapeutic approach in an AD mouse model [81]. A combination of AAV vectors and miRNA therapeutics has been demonstrated to reduce neuronal toxicity in primates [82]. Although miRNA-based BACE1 inhibition has been extensively investigated, the development of AAV-based delivery of miRNAs provides an attractive translatable therapy with continued efficacy and safety [83,84]. AAV-delivered miRNA gene therapies targeting BACE1 remain to be explored. Our data propose AAV-based delivery of miR-143-3p targeting BACE1 mRNA for sustained inhibition of BACE1 via a single injection. Nevertheless, delivery limitations of AAV need to be further overcome before translating to clinical practice, such as long-term safety issues, cell-specificity of the vector, dosage control, and feasibility of stereotaxic administration as a method of clinical delivery [45].

Emerging evidence suggests that disruption of miRNA homeostasis in the brain might drive or co-drive certain pathological cascades [85]. As naturally occurring molecules, miRNAs exert relatively weak effects on their targets and possess the mechanisms in place for downstream signaling [86,87,88]. Several miRNA-based therapeutics have entered clinical trials for patients suffering from a wide variety of diseases [85]. On the basis of our findings that miR-143-3p was downregulated in the hippocampus of AD brains, we developed miRNA-based therapeutics by stereotaxic injection of AAV to restore miR-143-3p levels in the hippocampus. In this study, our data suggest that inhibition of BACE1 by restoration of miR-143-3p in the hippocampus could improve the cognitive functions of AD model mice. By contrast, through intracerebroventricular injection of miR-132, another miRNA strongly decreased in AD, the hippocampal neurogenesis and memory deficits could be rescued after restoration in AD mouse models [89].

A recent study illustrating the regulatory effects of BACE1 in the hippocampal CA1 on learning and memory circuits not only highlights the critical physiological roles of BACE1 but also suggests that CA1 is a noteworthy subfield in the hippocampus modulating the cognitive circuit [22]. The CA1 hippocampal region is vulnerable to being affected in AD, mainly at early stages, and performs distinct memory functions [90,91,92]. Compared with other subfields in the hippocampus, such as CA3, DG, and subiculum, the highest number of Aβ plaques was observed in the CA1 subfield from demented AD individuals [93]. A recent study also showed that the number of CA1 neurons was dramatically decreased, and Aβ plaques were aggravated in the CA1 subfield in AD [58]. Hippocampal CA1 LTP was demonstrated to directly affect spatial learning and memory [57]. Our data revealed that BACE1 levels were upregulated with age in the hippocampal CA1 subfield in 5×FAD mice, and restoring miR-143-3p in this subfield could effectively reduce Aβ deposition along with improved cognitive functions. Consistent with our results, another study also observed the upregulation of BACE1 in the CA1 subfield in the AD mouse brain, utilizing a highly selective and sensitive two-photon ratiometric fluorescent probe for BACE1 determination in vivo [94]. Our work suggested that BACE1 levels were not obviously altered in the hippocampal CA3 subfield with age. This could be due to high background levels of BACE1 in the CA3 subfield of the hippocampus in mouse brains, which was also shown by previous studies [44,95]. Given that CA1 subfield serves a fundamental role in cognitive functions, the use of miR-143-3p, specific to the CA1 subfield, may contribute to the alleviation of cognitive deficits. Our data suggested that AAV-miR-143-3p expression was mainly localized to the CA1 subfield, but also affected other neighboring hippocampal subfields, which was attributed to the spreading nature of AAV. Nevertheless, the vector spread to the cortex was minimal. It would be beneficial to further explore refined engineering strategies to minimize off-target effects.

In addition, a unique phenotypic morphology of microglia after Bace1 deletion was reported, and an increase in IBA1-labeled microglia in close contact with amyloid plaques was observed [63]. Consistently, our results also showed that AAV-mediated miR-143-3p restoration in the brains of AD mice could regulate phagocytic microglia surrounding Aβ plaques, and the size of amyloid plaques was visibly decreased.

Our study still has some limitations. First, we were limited by the brain tissue samples of AD patients and controls. It is important to examine miR-143-3p and BACE1 levels in distinct regions of the hippocampus in human brains in future work. Second, whether and how miR-143-3p directly controls microglia remains to be illuminated in the following research. Future studies are warranted to identify the potential roles and mechanisms of miR-143-3p in Aβ phagocytosis. Third, further experiments involving both loss- and gain-of-function studies are needed to enhance the mechanistic findings of the study.

## 5. Conclusions

**Figure 9:**
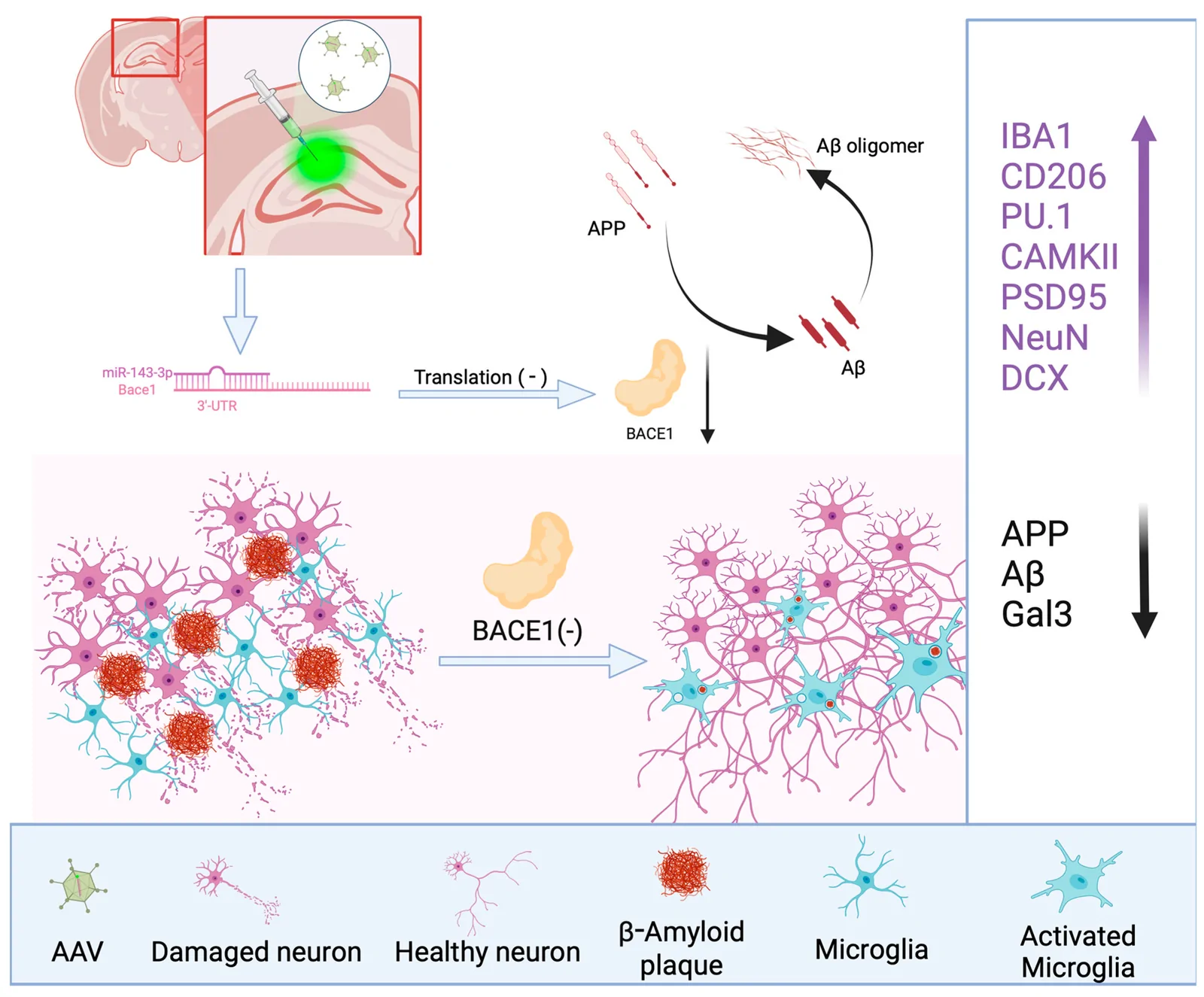
Schematic diagram summarizing the proposed regulatory mechanism of AAV-mediated restoration of miR-143-3p in the hippocampal CA1 area by targeting BACE1.

In summary, we demonstrate that in the hippocampus of AD individuals, the miR-143-3p levels are downregulated and inversely correlated with the expression of BACE1, which is identified as a direct target of miR-143-3p. Our data show that in 5×FAD model mice, BACE1 levels are upregulated with age in the CA1 subfield of the hippocampus. Through AAV-mediated miR-143-3p restoration in the hippocampal CA1 subfield of AD mice, cognitive performance is ameliorated, the expression of BACE1 and Aβ is reduced, the polarization of microglia toward the anti-inflammatory phenotype is promoted, neural-related genes, including Gal3, are modulated, and synaptic dysfunctions are rescued. Our mechanistic experiments reveal that restoring miR-143-3p in the hippocampal CA1 subfield decreases brain amyloid deposition and improves cognitive functions in an Alzheimer’s mouse model by suppressing BACE1 levels (Figure 9). Our data suggest that the AAV-mediated microRNA gene therapy strategy developed for spatial inhibition of BACE1 can effectively improve cognitive function in AD model mice, offering attractive options for AD therapeutics with long-term efficacy.

## Data Availability

All additional data supporting the findings are provided in the Supplementary Information and are available from the corresponding author upon reasonable request.

## Supplementary Materials

The following supporting information can be downloaded at: https://www.mdpi.com/article/10.3390/biom16081075/s1, Table S1: Characteristics of human brain samples for Western blot analyses; Table S2: Characteristics of human brain samples for immunofluorescence analyses; Figure S1: Quantification of BACE1 protein levels in the postmortem hippocampus of patients with AD and healthy individuals; Figure S2: The relative luciferase activity in N2a cells cotransfected with miR-143-3p mimics or controls and the pmirGLO vector containing the WT or MUT sequence; Figure S3: Quantification of BACE1 protein levels in SH-SY5Y APP cells and SH-SY5Y cells treated with miR-143-3p or controls; Figure S4: Representative immunostaining images of BACE1 and Aβ in the brains of AD mice and WT mice at various months of age, with nuclei stained with DAPI; Figure S5: Quantification of BACE1 levels in the brains of AD mice and WT mice at various months of age; Figure S6: Representative images showing the site of stereotaxical injection; Figure S7: Representative images of the lateral view demonstrating the site of stereotaxical injection; Figure S8: Quantification of AAV-miR-143-3p regional distribution in CA1, DG, and CA3 subfields; Figure S9: Quantification of AAV-miR-143-3p regional distribution in the hippocampus and cortex; Figure S10: Representative movement trajectories for AD mice in three groups in the OF test; Figure S11: Total distance for AD mice in three groups in the Y maze test; Figure S12: Representative images for AD mice of three groups in the nesting test; Figure S13: Representative immunostaining images of BACE1 in primary cultured neurons of 5×FAD model mice; Figure S14: Western blotting analysis of BACE1 in primary cultured neurons of 5×FAD model mice; Figure S15: Representative high-resolution images of IBA1 located around Aβ plaques in the brains of AD mice in three groups, with nuclei stained with DAPI; File S1: The original Western blot images.

## Abbreviations

The following abbreviations are used in this manuscript:

AD	Alzheimer’s disease
AAV	adeno-associated virus
ARIA	amyloid-related imaging abnormalities
APP	amyloid precursor protein
Aβ	beta-amyloid
BACE1	beta-site amyloid precursor protein-cleaving enzyme 1
CA1	cornu ammonis 1
DEG	differentially expressed gene
DG	dentate gyrus
FISH	fluorescence in situ hybridization
GO	gene ontology
IBA1	ionized calcium-binding adapter molecule 1
LOAD	late-onset Alzheimer’s disease
LTP	long-term potentiation
miRNA	microRNA
MWM	Morris water maze
MUT	mutant
NAD	nucleic acid drug
NeuN	neuronal nuclei
NOR	novel object recognition
OF	open field
PSD95	postsynaptic density 95
TNA	therapeutic nucleic acid
WT	wild-type

## References

[1] Scheltens P., De Strooper B., Kivipelto M., Holstege H., Chetelat G., Teunissen C.E., Cummings J., van der Flier W.M. (2021). Alzheimer’s disease. Lancet.

[2] Korczyn A.D., Grinberg L.T. (2024). Is Alzheimer disease a disease?. Nat. Rev. Neurol..

[3] Zheng Q., Wang X. (2025). Alzheimer’s disease: Insights into pathology, molecular mechanisms, and therapy. Protein Cell.

[4] Bosch M.E., Dodiya H.B., Michalkiewicz J., Lee C., Shaik S.M., Weigle I.Q., Zhang C., Osborn J., Nambiar A., Patel P. (2024). Sodium oligomannate alters gut microbiota, reduces cerebral amyloidosis and reactive microglia in a sex-specific manner. Mol. Neurodegener..

[5] Pawar S., Rauf M.A., Abdelhady H., Iyer A.K. (2025). Tau-targeting nanoparticles for treatment of Alzheimer’s disease. Exploration.

[6] van Dyck C.H., Swanson C.J., Aisen P., Bateman R.J., Chen C., Gee M., Kanekiyo M., Li D., Reyderman L., Cohen S. (2023). Lecanemab in Early Alzheimer’s Disease. N. Engl. J. Med..

[7] Rezai A.R., D’Haese P.F., Finomore V., Carpenter J., Ranjan M., Wilhelmsen K., Mehta R.I., Wang P., Najib U., Vieira Ligo Teixeira C. (2024). Ultrasound Blood-Brain Barrier Opening and Aducanumab in Alzheimer’s Disease. N. Engl. J. Med..

[8] Sevigny J., Chiao P., Bussiere T., Weinreb P.H., Williams L., Maier M., Dunstan R., Salloway S., Chen T., Ling Y. (2016). The antibody aducanumab reduces Abeta plaques in Alzheimer’s disease. Nature.

[9] Budd Haeberlein S., Aisen P.S., Barkhof F., Chalkias S., Chen T., Cohen S., Dent G., Hansson O., Harrison K., von Hehn C. (2022). Two Randomized Phase 3 Studies of Aducanumab in Early Alzheimer’s Disease. J. Prev. Alzheimer’s Dis..

[10] Sims J.R., Zimmer J.A., Evans C.D., Lu M., Ardayfio P., Sparks J., Wessels A.M., Shcherbinin S., Wang H., Monkul Nery E.S. (2023). Donanemab in Early Symptomatic Alzheimer Disease: The TRAILBLAZER-ALZ 2 Randomized Clinical Trial. JAMA.

[11] Jucker M., Walker L.C. (2023). Alzheimer’s disease: From immunotherapy to immunoprevention. Cell.

[12] Zhang Y., Chen H., Li R., Sterling K., Song W. (2023). Amyloid beta-based therapy for Alzheimer’s disease: Challenges, successes and future. Signal Transduct. Target. Ther..

[13] Reardon S. (2023). FDA approves Alzheimer’s drug lecanemab amid safety concerns. Nature.

[14] Yang L.B., Lindholm K., Yan R., Citron M., Xia W., Yang X.L., Beach T., Sue L., Wong P., Price D. (2003). Elevated beta-secretase expression and enzymatic activity detected in sporadic Alzheimer disease. Nat. Med..

[15] Ghosh A.K., Osswald H.L. (2014). BACE1 (beta-secretase) inhibitors for the treatment of Alzheimer’s disease. Chem. Soc. Rev..

[16] Bao H., Shen Y. (2023). Unmasking BACE1 in aging and age-related diseases. Trends Mol. Med..

[17] Ding X., Hu Y., Feng X., Wang Z., Song Q., Dai C., Yang B., Fu X., Sun D., Fan C. (2025). Enhanced Blood-Brain Barrier Penetrability of BACE1 SiRNA-Loaded Prussian Blue Nanocomplexes for Alzheimer’s Disease Synergy Therapy. Exploration.

[18] Watkins E.A., Vassar R. (2024). BACE Inhibitor Clinical Trials for Alzheimer’s Disease. J. Alzheimer’s Dis..

[19] Tariot P.N., Riviere M.E., Salloway S., Burns J.M., Snaedal J.G., Borowsky B., Lopez C.L., Liu F., Rouzade-Dominguez M.L., Cazorla P. (2024). Reversibility of cognitive worsening observed with BACE inhibitor umibecestat in the Alzheimer’s Prevention Initiative (API) Generation Studies. Alzheimer’s Dement..

[20] Ou-Yang M.H., Kurz J.E., Nomura T., Popovic J., Rajapaksha T.W., Dong H., Contractor A., Chetkovich D.M., Tourtellotte W.G., Vassar R. (2018). Axonal organization defects in the hippocampus of adult conditional BACE1 knockout mice. Sci. Transl. Med..

[21] Das B., Singh N., Yao A.Y., Zhou J., He W., Hu X., Yan R. (2021). BACE1 controls synaptic function through modulating release of synaptic vesicles. Mol. Psychiatry.

[22] Xiao X., Wang X., Zhu K., Li L., He Y., Zhang J., Li L., Hu H., Cui Y., Zhang J. (2023). BACE1 in PV interneuron tunes hippocampal CA1 local circuits and resets priming of fear memory extinction. Mol. Psychiatry.

[23] Sun X., Setrerrahmane S., Li C., Hu J., Xu H. (2024). Nucleic acid drugs: Recent progress and future perspectives. Signal Transduct. Target. Ther..

[24] Li Y., Chen S., Rao H., Cui S., Chen G. (2025). MicroRNA Gets a Mighty Award. Adv. Sci..

[25] Chen S., Chen Q., You X., Zhou Z., Kong N., Ambrosio F., Cao Y., Abdi R., Tao W. (2025). Using RNA therapeutics to promote healthy aging. Nat. Aging.

[26] Jurj A., Dragomir M.P., Li Z., Calin G.A. (2026). MicroRNAs in oncology: A translational perspective in the era of AI. Nat. Rev. Clin. Oncol..

[27] Xu L., Shao Z., Fang X., Xin Z., Zhao S., Zhang H., Zhang Y., Zheng W., Yu X., Zhang Z. (2025). Exploring precision treatments in immune-mediated inflammatory diseases: Harnessing the infinite potential of nucleic acid delivery. Exploration.

[28] Chakravarthy M., Chen S., Dodd P.R., Veedu R.N. (2017). Nucleic Acid-Based Theranostics for Tackling Alzheimer’s Disease. Theranostics.

[29] Afonin K.A., Dobrovolskaia M.A., Church G., Bathe M. (2020). Opportunities, Barriers, and a Strategy for Overcoming Translational Challenges to Therapeutic Nucleic Acid Nanotechnology. ACS Nano.

[30] Gupta A., Andresen J.L., Manan R.S., Langer R. (2021). Nucleic acid delivery for therapeutic applications. Adv. Drug Deliv. Rev..

[31] Kumar R., Santa Chalarca C.F., Bockman M.R., Bruggen C.V., Grimme C.J., Dalal R.J., Hanson M.G., Hexum J.K., Reineke T.M. (2021). Polymeric Delivery of Therapeutic Nucleic Acids. Chem. Rev..

[32] Sanadgol N., Abedi M., Hashemzaei M., Kamran Z., Khalseh R., Beyer C., Voelz C. (2025). Exosomes as nanocarriers for brain-targeted delivery of therapeutic nucleic acids: Advances and challenges. J. Nanobiotechnol..

[33] Wang L., Shui X., Diao Y., Chen D., Zhou Y., Lee T.H. (2023). Potential Implications of miRNAs in the Pathogenesis, Diagnosis, and Therapeutics of Alzheimer’s Disease. Int. J. Mol. Sci..

[34] Callaway E., Sanderson K. (2024). Medicine Nobel awarded for gene-regulating ‘microRNAs’. Nature.

[35] Sun Z., Kwon J.S., Ren Y., Chen S., Walker C.K., Lu X., Cates K., Karahan H., Sviben S., Fitzpatrick J.A.J. (2024). Modeling late-onset Alzheimer’s disease neuropathology via direct neuronal reprogramming. Science.

[36] Dong H., Li J., Huang L., Chen X., Li D., Wang T., Hu C., Xu J., Zhang C., Zen K. (2015). Serum MicroRNA Profiles Serve as Novel Biomarkers for the Diagnosis of Alzheimer’s Disease. Dis. Markers.

[37] Cheng L., Doecke J.D., Sharples R.A., Villemagne V.L., Fowler C.J., Rembach A., Martins R.N., Rowe C.C., Macaulay S.L., Masters C.L. (2015). Prognostic serum miRNA biomarkers associated with Alzheimer’s disease shows concordance with neuropsychological and neuroimaging assessment. Mol. Psychiatry.

[38] Choi W., Yeom S.Y., Kim J., Jung S., Jung S., Shim T.S., Kim S.K., Kang J.Y., Lee S.H., Cho I.J. (2018). Hydrogel micropost-based qPCR for multiplex detection of miRNAs associated with Alzheimer’s disease. Biosens. Bioelectron..

[39] Ebrahimi A., Ravan H., Mehrabani M. (2020). Multiplex monitoring of Alzheimer associated miRNAs based on the modular logic circuit operation and doping of catalytic hairpin assembly. Biosens. Bioelectron..

[40] Jia L., Zhu M., Yang J., Pang Y., Wang Q., Li Y., Li T., Li F., Wang Q., Li Y. (2021). Prediction of P-tau/Abeta42 in the cerebrospinal fluid with blood microRNAs in Alzheimer’s disease. BMC Med..

[41] Li D., Chen Y., Zhang T., Lv Z., Zhang L., Li X., Zhang A. (2023). Profiling microRNA from peripheral blood mononuclear cells in early-onset familial Alzheimer’s disease. Neuroreport.

[42] Depp C., Sun T., Sasmita A.O., Spieth L., Berghoff S.A., Nazarenko T., Overhoff K., Steixner-Kumar A.A., Subramanian S., Arinrad S. (2023). Myelin dysfunction drives amyloid-beta deposition in models of Alzheimer’s disease. Nature.

[43] Oakley H., Cole S.L., Logan S., Maus E., Shao P., Craft J., Guillozet-Bongaarts A., Ohno M., Disterhoft J., Van Eldik L. (2006). Intraneuronal beta-amyloid aggregates, neurodegeneration, and neuron loss in transgenic mice with five familial Alzheimer’s disease mutations: Potential factors in amyloid plaque formation. J. Neurosci..

[44] Zhao J., Fu Y., Yasvoina M., Shao P., Hitt B., O’Connor T., Logan S., Maus E., Citron M., Berry R. (2007). Beta-site amyloid precursor protein cleaving enzyme 1 levels become elevated in neurons around amyloid plaques: Implications for Alzheimer’s disease pathogenesis. J. Neurosci..

[45] Wang J.H., Gessler D.J., Zhan W., Gallagher T.L., Gao G. (2024). Adeno-associated virus as a delivery vector for gene therapy of human diseases. Signal Transduct. Target. Ther..

[46] Zhou Y., Guo Y., Chen L., Zhang X., Wu W., Yang Z., Li X., Wang Y., Hu Z., Wang Z. (2022). Co-delivery of phagocytosis checkpoint and STING agonist by a Trojan horse nanocapsule for orthotopic glioma immunotherapy. Theranostics.

[47] Zhou Y., Wang L., Chen L.F., Wu W., Yang Z.M., Wang Y.Z., Wang A.Q., Jiang S.J., Qin X.Z., Ye Z.C. (2023). Glioblastoma cell-derived exosomes functionalized with peptides as efficient nanocarriers for synergistic chemotherapy of glioblastoma with improved biosafety. Nano Res..

[48] Zhou Y., Cai G., Wang Y., Guo Y., Yang Z., Wang A., Chen Y., Li X., Chen X., Hu Z. (2024). Microarray Chip-Based High-Throughput Screening of Neurofilament Light Chain Self-Assembling Peptide for Noninvasive Monitoring of Alzheimer’s Disease. ACS Nano.

[49] Kim B.M., You M.H., Chen C.H., Suh J., Tanzi R.E., Ho Lee T. (2016). Inhibition of death-associated protein kinase 1 attenuates the phosphorylation and amyloidogenic processing of amyloid precursor protein. Hum. Mol. Genet..

[50] Guo S., Li Y., Wei B., Liu W., Li R., Cheng W., Zhang X., He X., Li X., Duan C. (2020). Tim-3 deteriorates neuroinflammatory and neurocyte apoptosis after subarachnoid hemorrhage through the Nrf2/HMGB1 signaling pathway in rats. Aging.

[51] Zhang C., Chen Y., Guo J., Zeng Y., Sun X., Yang W., Wang J., Wang K., Wang D., Qi X. (2026). Ccl12 coordinates immune-neural crosstalk to promote adipose sympathetic remodeling after burn trauma. Cell Rep..

[52] Shui X., Zheng X., Wu J., Zhang M., Kim G., Chen R., Peng L., Wang Z., Zheng Y., Zhang L. (2025). Death-associated protein kinase 1-dependent SENP1 degradation increases tau SUMOylation and leads to cognitive dysfunction in a mouse model for tauopathy. Mol. Neurodegener..

[53] Hampel H., Vassar R., De Strooper B., Hardy J., Willem M., Singh N., Zhou J., Yan R., Vanmechelen E., De Vos A. (2021). The beta-Secretase BACE1 in Alzheimer’s Disease. Biol. Psychiatry.

[54] Wang L., Shui X., Mei Y., Xia Y., Lan G., Hu L., Zhang M., Gan C.L., Li R., Tian Y. (2022). miR-143-3p Inhibits Aberrant Tau Phosphorylation and Amyloidogenic Processing of APP by Directly Targeting DAPK1 in Alzheimer’s Disease. Int. J. Mol. Sci..

[55] El Fatimy R., Zhang Y., Deforzh E., Ramadas M., Saravanan H., Wei Z., Rabinovsky R., Teplyuk N.M., Uhlmann E.J., Krichevsky A.M. (2022). A nuclear function for an oncogenic microRNA as a modulator of snRNA and splicing. Mol. Cancer.

[56] Uytiepo M., Zhu Y., Bushong E., Chou K., Polli F.S., Zhao E., Kim K.Y., Luu D., Chang L., Yang D. (2025). Synaptic architecture of a memory engram in the mouse hippocampus. Science.

[57] Xia L., Li J., Pang Y., Dai C., Xu M., Du Y., Tian Q., Yi L., Wu B., Chen M. (2025). Disruption of BAG3-mediated BACE1 stabilization alleviates neuropathology and memory deficits in a mouse model of Alzheimer’s disease. Sci. Adv..

[58] Wang P., Han L., Wang L., Tao Q., Guo Z., Luo T., He Y., Xu Z., Yu J., Liu Y. (2025). Molecular pathways and diagnosis in spatially resolved Alzheimer’s hippocampal atlas. Neuron.

[59] Wang Q., Ding S.L., Li Y., Royall J., Feng D., Lesnar P., Graddis N., Naeemi M., Facer B., Ho A. (2020). The Allen Mouse Brain Common Coordinate Framework: A 3D Reference Atlas. Cell.

[60] Li W.B., Xu L.L., Wang S.L., Wang Y.Y., Pan Y.C., Shi L.Q., Guo D.S. (2024). Co-Assembled Nanoparticles toward Multi-Target Combinational Therapy of Alzheimer’s Disease by Making Full Use of Molecular Recognition and Self-Assembly. Adv. Mater..

[61] Dai X., Lin A., Zhuang L., Zeng Q., Cai L., Wei Y., Liang H., Gao W., Zhang J., Chen X. (2024). Targeting SIK3 to modulate hippocampal synaptic plasticity and cognitive function by regulating the transcription of HDAC4 in a mouse model of Alzheimer’s disease. Neuropsychopharmacology.

[62] Xie Y., Liu J., Hou Z., Wang H., Liu K., Chen X., Fan Z., Li D., Li C., Pan Y. (2025). CD4-Derived Double-Negative T Cells Ameliorate Alzheimer’s Disease-Like Phenotypes in the 5xFAD Mouse Model. CNS Neurosci. Ther..

[63] Singh N., Das B., Zhou J., Hu X., Yan R. (2022). Targeted BACE-1 inhibition in microglia enhances amyloid clearance and improved cognitive performance. Sci. Adv..

[64] Boza-Serrano A., Ruiz R., Sanchez-Varo R., Garcia-Revilla J., Yang Y., Jimenez-Ferrer I., Paulus A., Wennstrom M., Vilalta A., Allendorf D. (2019). Galectin-3, a novel endogenous TREM2 ligand, detrimentally regulates inflammatory response in Alzheimer’s disease. Acta Neuropathol..

[65] Tao C.C., Cheng K.M., Ma Y.L., Hsu W.L., Chen Y.C., Fuh J.L., Lee W.J., Chao C.C., Lee E.H.Y. (2020). Galectin-3 promotes Abeta oligomerization and Abeta toxicity in a mouse model of Alzheimer’s disease. Cell Death Differ..

[66] Boza-Serrano A., Vrillon A., Minta K., Paulus A., Camprubi-Ferrer L., Garcia M., Andreasson U., Antonell A., Wennstrom M., Gouras G. (2022). Galectin-3 is elevated in CSF and is associated with Abeta deposits and tau aggregates in brain tissue in Alzheimer’s disease. Acta Neuropathol..

[67] Kim B., Dabin L.C., Tate M.D., Karahan H., Sharify A.D., Acri D.J., Al-Amin M.M., Philtjens S., Smith D.C., Wijeratne H.R.S. (2024). Effects of SPI1-mediated transcriptome remodeling on Alzheimer’s disease-related phenotypes in mouse models of Abeta amyloidosis. Nat. Commun..

[68] Shao J., Youngblood H., Yang L. (2025). Targeting SPI1 to mitigate amyloid-beta pathology in Alzheimer’s disease. J. Alzheimer’s Dis..

[69] Serrano-Pozo A., Frosch M.P., Masliah E., Hyman B.T. (2011). Neuropathological alterations in Alzheimer disease. Cold Spring Harb. Perspect. Med..

[70] Dejanovic B., Sheng M., Hanson J.E. (2024). Targeting synapse function and loss for treatment of neurodegenerative diseases. Nat. Rev. Drug Discov..

[71] Long J.M., Holtzman D.M. (2019). Alzheimer Disease: An Update on Pathobiology and Treatment Strategies. Cell.

[72] Morris J.C., Weiner M., Xiong C., Beckett L., Coble D., Saito N., Aisen P.S., Allegri R., Benzinger T.L.S., Berman S.B. (2022). Autosomal dominant and sporadic late onset Alzheimer’s disease share a common in vivo pathophysiology. Brain.

[73] Zhang J., Zhang Y., Wang J., Xia Y., Zhang J., Chen L. (2024). Recent advances in Alzheimer’s disease: Mechanisms, clinical trials and new drug development strategies. Signal Transduct. Target. Ther..

[74] Jia J., Ning Y., Chen M., Wang S., Yang H., Li F., Ding J., Li Y., Zhao B., Lyu J. (2024). Biomarker Changes during 20 Years Preceding Alzheimer’s Disease. N. Engl. J. Med..

[75] Singh N., Benoit M.R., Zhou J., Das B., Davila-Velderrain J., Kellis M., Tsai L.H., Hu X., Yan R. (2022). BACE-1 inhibition facilitates the transition from homeostatic microglia to DAM-1. Sci. Adv..

[76] Muller S.A., Shmueli M.D., Feng X., Tushaus J., Schumacher N., Clark R., Smith B.E., Chi A., Rose-John S., Kennedy M.E. (2023). The Alzheimer’s disease-linked protease BACE1 modulates neuronal IL-6 signaling through shedding of the receptor gp130. Mol. Neurodegener..

[77] Zhou J., Singh N., Galske J., Hudobenko J., Hu X., Yan R. (2023). BACE1 regulates expression of Clusterin in astrocytes for enhancing clearance of beta-amyloid peptides. Mol. Neurodegener..

[78] Sasmita A.O., Depp C., Nazarenko T., Sun T., Siems S.B., Ong E.C., Nkeh Y.B., Bohler C., Yu X., Bues B. (2024). Oligodendrocytes produce amyloid-beta and contribute to plaque formation alongside neurons in Alzheimer’s disease model mice. Nat. Neurosci..

[79] Bi D., Bao H., Yang X., Wu Z., Yang X., Xu G., Liu X., Wan Z., Liu J., He J. (2025). BACE1-dependent cleavage of GABA(A) receptor contributes to neural hyperexcitability and disease progression in Alzheimer’s disease. Neuron.

[80] Imran S., Patel M., Noroozifar M., Kerman K. (2026). Recent advances towards BACE1 drug discovery and therapeutics design. RSC Med. Chem..

[81] Marino M., Zhou L., Rincon M.Y., Callaerts-Vegh Z., Verhaert J., Wahis J., Creemers E., Yshii L., Wierda K., Saito T. (2022). AAV-mediated delivery of an anti-BACE1 VHH alleviates pathology in an Alzheimer’s disease model. EMBO Mol. Med..

[82] Hordeaux J., Buza E.L., Jeffrey B., Song C., Jahan T., Yuan Y., Zhu Y., Bell P., Li M., Chichester J.A. (2020). MicroRNA-mediated inhibition of transgene expression reduces dorsal root ganglion toxicity by AAV vectors in primates. Sci. Transl. Med..

[83] Segur-Bailach E., Mateu-Bosch A., Bofill-De Ros X., Pares M., da Silva Buttkus P., Rathkolb B., Gailus-Durner V., Hrabe de Angelis M., Moeini P., Gonzalez-Aseguinolaza G. (2025). Therapeutic AASS inhibition by AAV-miRNA rescues glutaric aciduria type I severe phenotype in mice. Mol. Ther..

[84] Tomassy G.S., Fan W., Cao S., Luo Z., Magli A., Zhang T., Boyle K., Jackson R., Richards B., Liu D. (2025). Development of an AAV-delivered microRNA gene therapy for myotonic dystrophy type 1. Mol. Ther..

[85] Walgrave H., Zhou L., De Strooper B., Salta E. (2021). The promise of microRNA-based therapies in Alzheimer’s disease: Challenges and perspectives. Mol. Neurodegener..

[86] Gebert L.F.R., MacRae I.J. (2019). Regulation of microRNA function in animals. Nat. Rev. Mol. Cell Biol..

[87] Winkle M., El-Daly S.M., Fabbri M., Calin G.A. (2021). Noncoding RNA therapeutics—Challenges and potential solutions. Nat. Rev. Drug Discov..

[88] Diener C., Keller A., Meese E. (2022). Emerging concepts of miRNA therapeutics: From cells to clinic. Trends Genet..

[89] Walgrave H., Balusu S., Snoeck S., Vanden Eynden E., Craessaerts K., Thrupp N., Wolfs L., Horre K., Fourne Y., Ronisz A. (2021). Restoring miR-132 expression rescues adult hippocampal neurogenesis and memory deficits in Alzheimer’s disease. Cell Stem Cell.

[90] Pini L., Pievani M., Bocchetta M., Altomare D., Bosco P., Cavedo E., Galluzzi S., Marizzoni M., Frisoni G.B. (2016). Brain atrophy in Alzheimer’s Disease and aging. Ageing Res. Rev..

[91] Furcila D., DeFelipe J., Alonso-Nanclares L. (2018). A Study of Amyloid-beta and Phosphotau in Plaques and Neurons in the Hippocampus of Alzheimer’s Disease Patients. J. Alzheimer’s Dis..

[92] Dong C., Madar A.D., Sheffield M.E.J. (2021). Distinct place cell dynamics in CA1 and CA3 encode experience in new environments. Nat. Commun..

[93] Furcila D., Dominguez-Alvaro M., DeFelipe J., Alonso-Nanclares L. (2019). Subregional Density of Neurons, Neurofibrillary Tangles and Amyloid Plaques in the Hippocampus of Patients with Alzheimer’s Disease. Front. Neuroanat..

[94] Ge L., Liu Z., Tian Y. (2020). A novel two-photon ratiometric fluorescent probe for imaging and sensing of BACE1 in different regions of AD mouse brain. Chem. Sci..

[95] Zhang X.M., Cai Y., Xiong K., Cai H., Luo X.G., Feng J.C., Clough R.W., Struble R.G., Patrylo P.R., Yan X.X. (2009). Beta-secretase-1 elevation in transgenic mouse models of Alzheimer’s disease is associated with synaptic/axonal pathology and amyloidogenesis: Implications for neuritic plaque development. Eur. J. Neurosci..

