# Supplementary material for: Development of a Novel AAV-Mediated microRNA Gene Therapy for Spatial Suppression of BACE1 to Improve Cognitive Function in Alzheimer’s Disease Model Mice
Source: Biomolecules. 2026 Jul 23;16(8):1075. doi: 10.3390/biom16081075 (PMC13509884; doi:10.3390/biom16081075)
Supplement: Supplementary file 1 [file biomolecules-16-01075-s001.zip › Supplementary Material S1 (Table S1 and S2).pdf]

Table S1. Characteristics of human brain samples for Western blot analyses.

NPDx	Gender	Age	Diagnosis	PMI (h)	B&B	CERAD
Control	F	66	/	25.00	NA	A
Control	F	68	/	NA	I	A
Control	M	84	/	16.00	I	A
Control	F	83	/	29.40	I	A
AD	F	83	AD	22.92	VI	C
AD	F	80	AD	22.00	VI	C
AD	F	76	AD	21.00	III	C
AD	F	80	AD	19.00	VI	C
AD	F	92	AD	24.00	VI	C
AD	F	84	AD	18.33	VI	C

Abbreviations: B&B (out of VI), Braak and Braak stage of AD; CERAD, Consortium to Establish a Registry for Alzheimer's disease; F, female; NPDx, neuropsychological diagnosis; PMI, postmortem interval.

Table S2. Characteristics of human brain samples for immunofluorescence analyses.

NPDx	Gender	Age	Diagnosis	PMI (h)	B&B	CERAD
Control	F	66	/	25.00	NA	A
Control	F	63	/	26.83	NA	A
Control	F	79	/	17.58	NA	A
Control	F	82	/	24.42	NA	A
AD	F	83	AD	22.92	VI	C
AD	M	86	AD	17.58	V	C
AD	F	82	AD	16.83	VI	C
AD	F	84	AD	18.33	VI	C

Abbreviations: B&B (out of VI), Braak and Braak stage of AD; CERAD, Consortium to Establish a Registry for Alzheimer's disease; F, female; NPDx, neuropsychological diagnosis; PMI, postmortem interval.
