# Supplementary material for: Development of a Novel AAV-Mediated microRNA Gene Therapy for Spatial Suppression of BACE1 to Improve Cognitive Function in Alzheimer’s Disease Model Mice
Source: Biomolecules. 2026 Jul 23;16(8):1075. doi: 10.3390/biom16081075 (PMC13509884; doi:10.3390/biom16081075)
Supplement: Supplementary file 1 [file biomolecules-16-01075-s001.zip › Supplementary Material S2 (Figure S1 to S15).pdf]

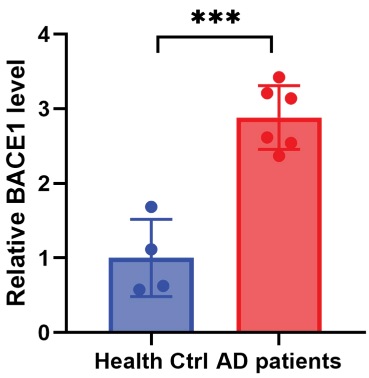

Figure S1. Quantification of BACE1 protein levels in the postmortem hippocampus of patients with AD and healthy individuals.

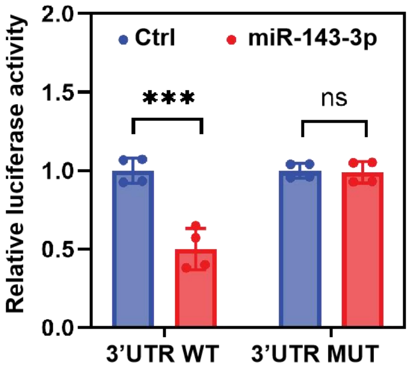

Figure S2. The relative luciferase activity in N2a cells cotransfected with miR-143-3p mimics or controls and the pmirGLO vector containing the WT or MUT sequence.

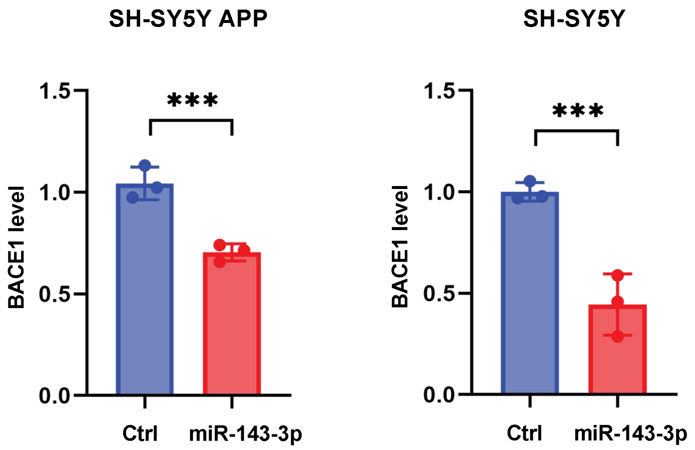

Figure S3. Quantification of BACE1 protein levels in SH-SY5Y APP cells and SH-SY5Y cells treated with miR-143-3p or controls.

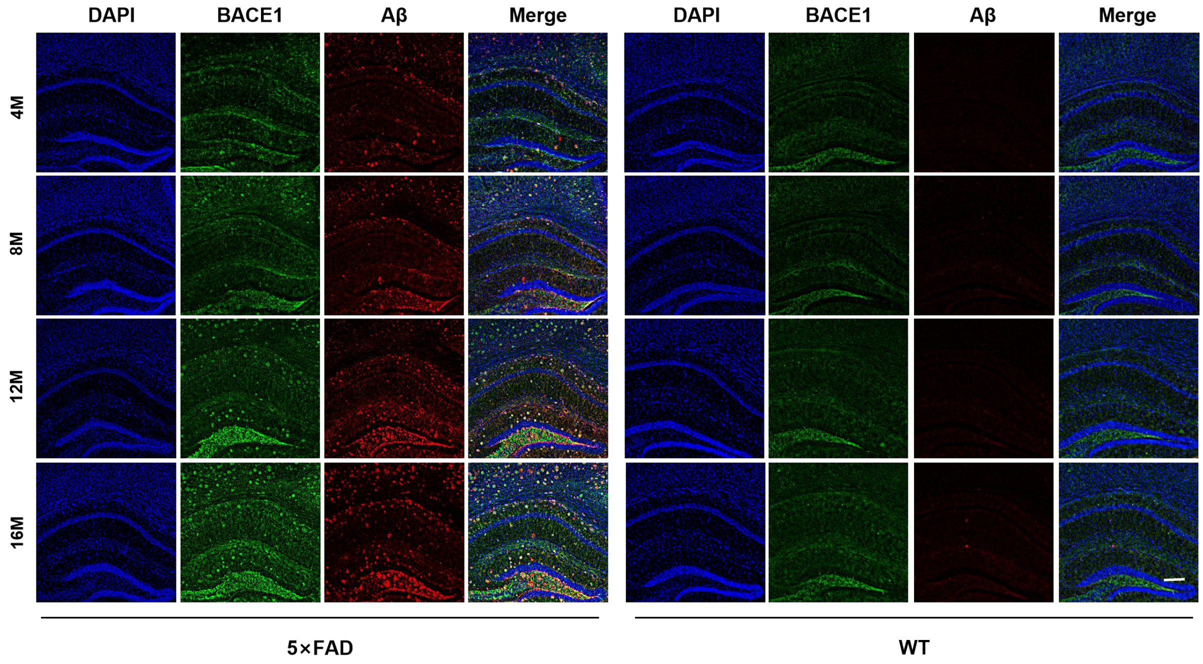

Figure S4. Representative immunostaining images of BACE1 and Aβ in the brains of AD mice and WT mice at various months of age, with nuclei stained with DAPI. Scale bar: 200 μm.

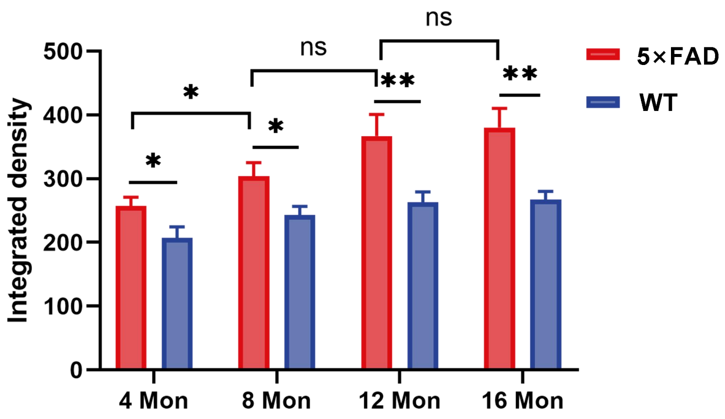

Figure S5. Quantification of BACE1 levels in the brains of AD mice and WT mice at various months of age.

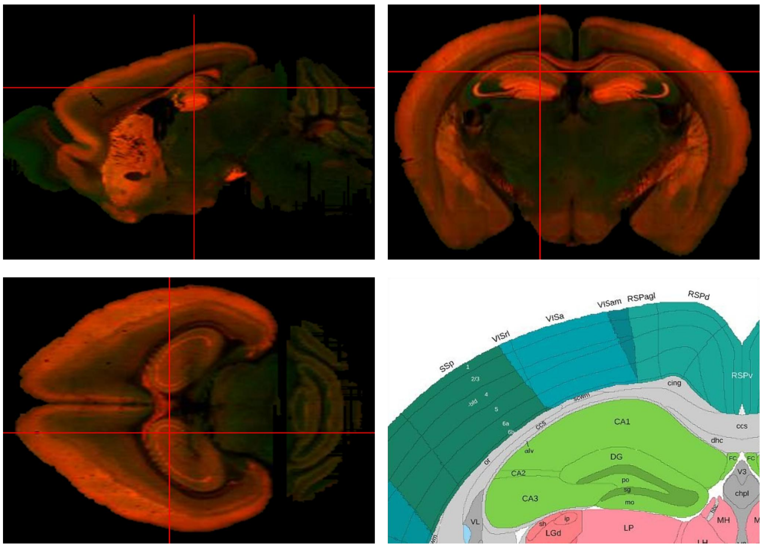

Figure S6. Representative images showing the site of stereotaxical injection.

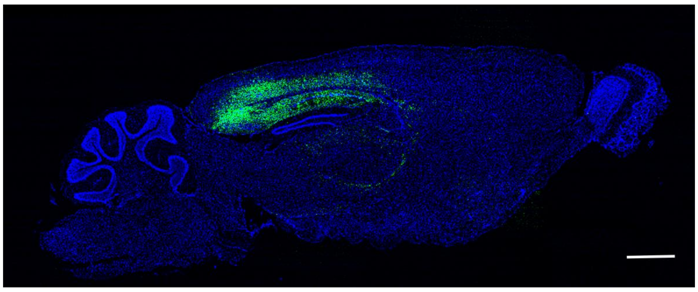

Figure S7. Representative images of the lateral view demonstrating the site of stereotaxical injection.

Scale bar: 150 mm.

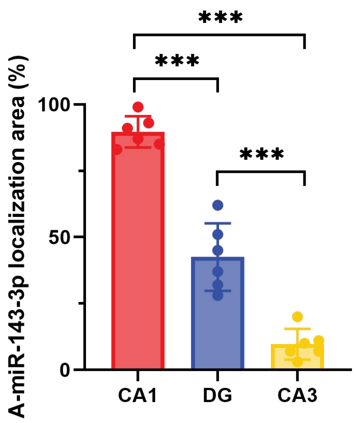

Figure S8. Quantification of AAV-miR-143-3p regional distribution in CA1, DG, and CA3 subfields.

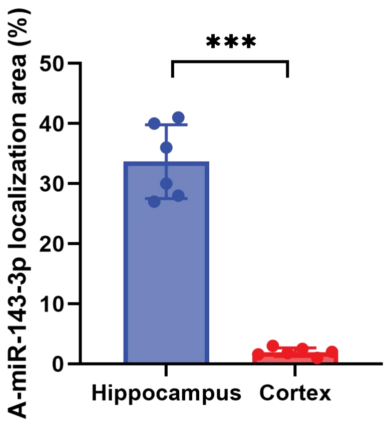

Figure S9. Quantification of AAV-miR-143-3p regional distribution in the hippocampus and cortex.

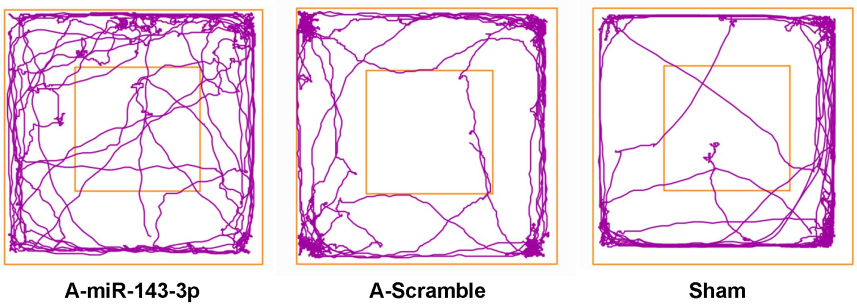

Figure S10. Representative movement trajectories for AD mice in three groups in the OF test.

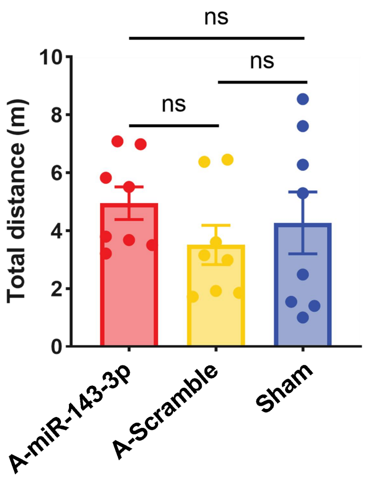

Figure S11. Total distance for AD mice in three groups in the Y maze test. ns, not significant.

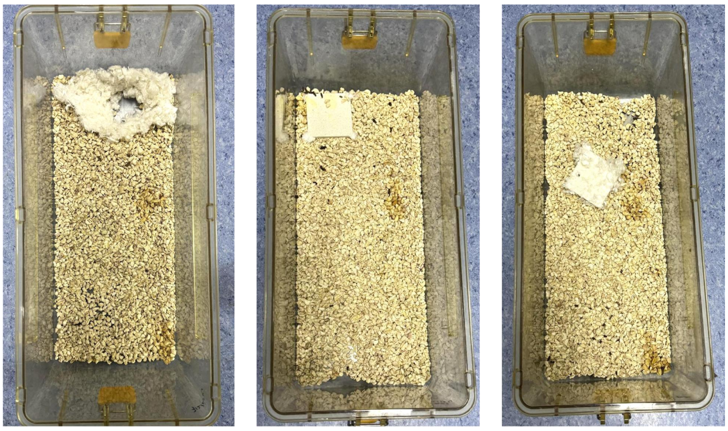

Figure S12. Representative images for AD mice of three groups in the nesting test.

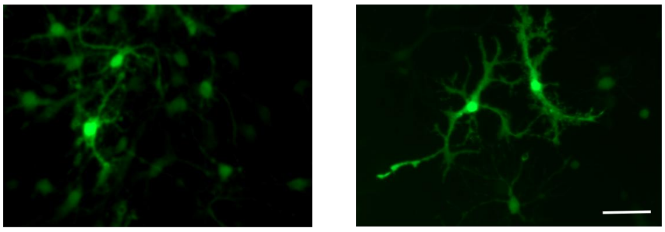

A-miR-143-3p **A-Scramble**

Figure S13. Representative immunostaining images of BACE1 in primary cultured neurons of 5×FAD model mice.

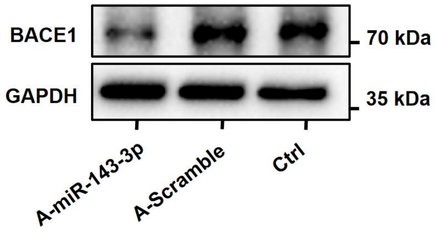

Figure S14. Western blotting analysis of BACE1 in primary cultured neurons of 5×FAD model mice.

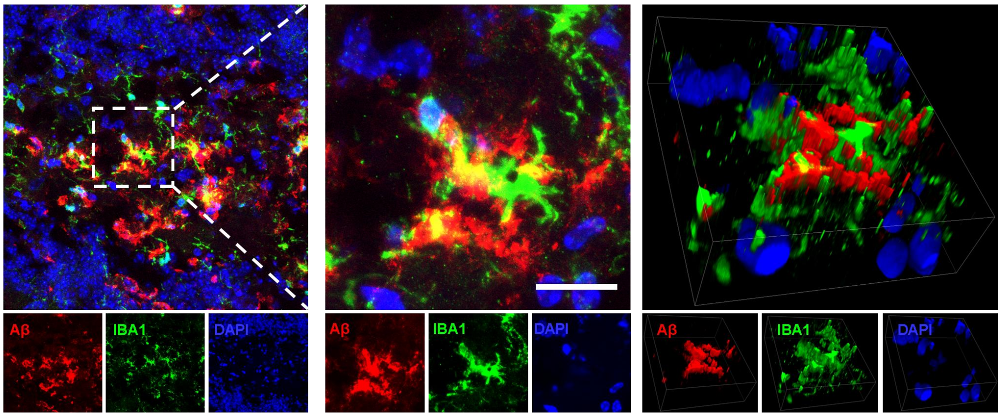

Figure S15. Representative high-resolution images of IBA1 located around Aβ plaques in the brains of AD mice in three groups, with nuclei stained with DAPI. Scale bar: 25 μm.
