# Supplementary material for: Development of a Novel AAV-Mediated microRNA Gene Therapy for Spatial Suppression of BACE1 to Improve Cognitive Function in Alzheimer’s Disease Model Mice
Source: Biomolecules. 2026 Jul 23;16(8):1075. doi: 10.3390/biom16081075 (PMC13509884; doi:10.3390/biom16081075)
Supplement: Supplementary file 1 [file biomolecules-16-01075-s001.zip › File S1. The orginal Western blot images.pdf]

a

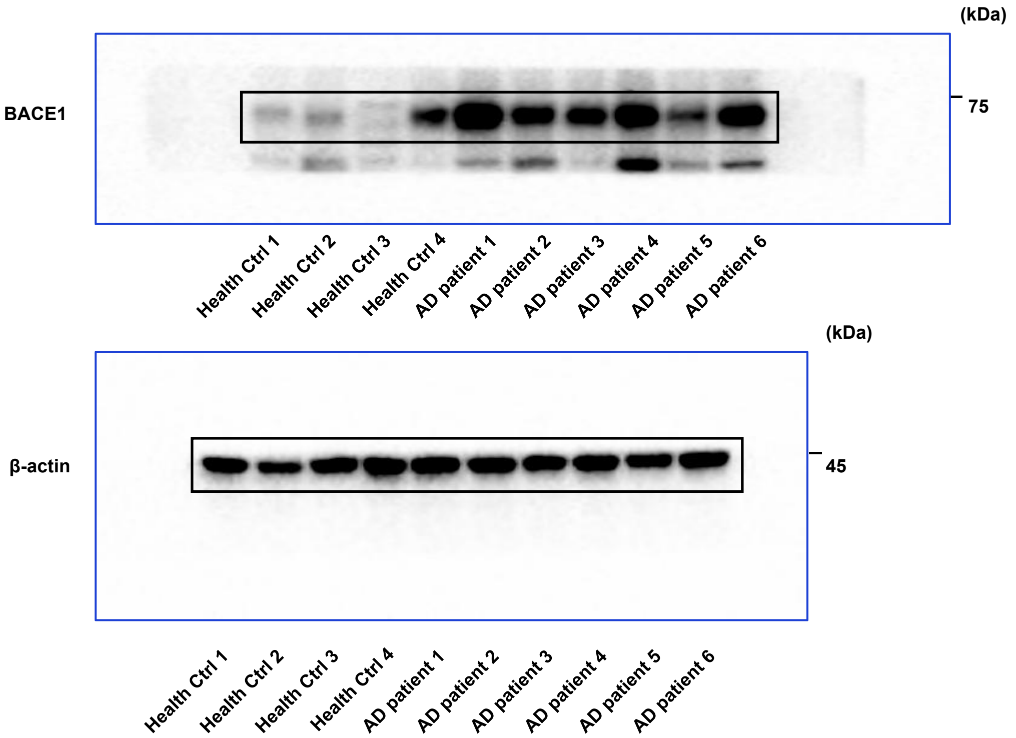
(kDa)
BACE1
75
Health Ctrl 1
Health Ctrl 2
Health Ctrl 3
Health Ctrl 4
AD patient 1
AD patient 2
AD patient 3
AD patient 4
AD patient 5
AD patient 6
(kDa)
β-actin
45
Health Ctrl 1
Health Ctrl 2
Health Ctrl 3
Health Ctrl 4
AD patient 1
AD patient 2
AD patient 3
AD patient 4
AD patient 5
AD patient 6

h

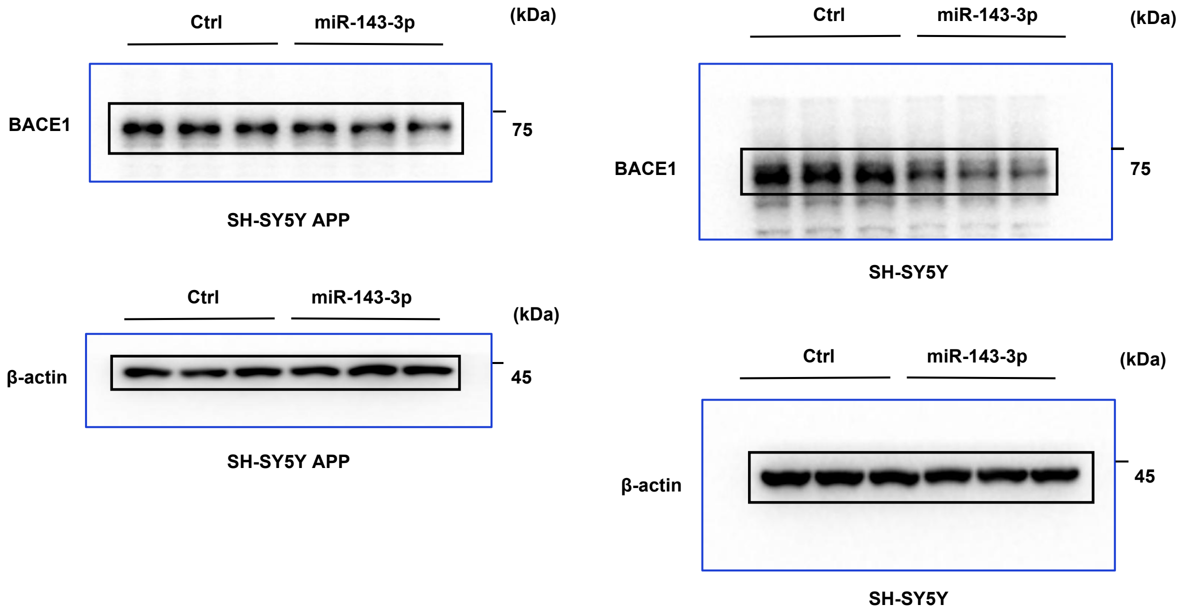
Ctrl
miR-143-3p
(kDa)
BACE1
75
SH-SY5Y APP
Ctrl
miR-143-3p
(kDa)
β-actin
45
SH-SY5Y APP
Ctrl
miR-143-3p
(kDa)
BACE1
75
SH-SY5Y
Ctrl
miR-143-3p
(kDa)
β-actin
45
SH-SY5Y

a

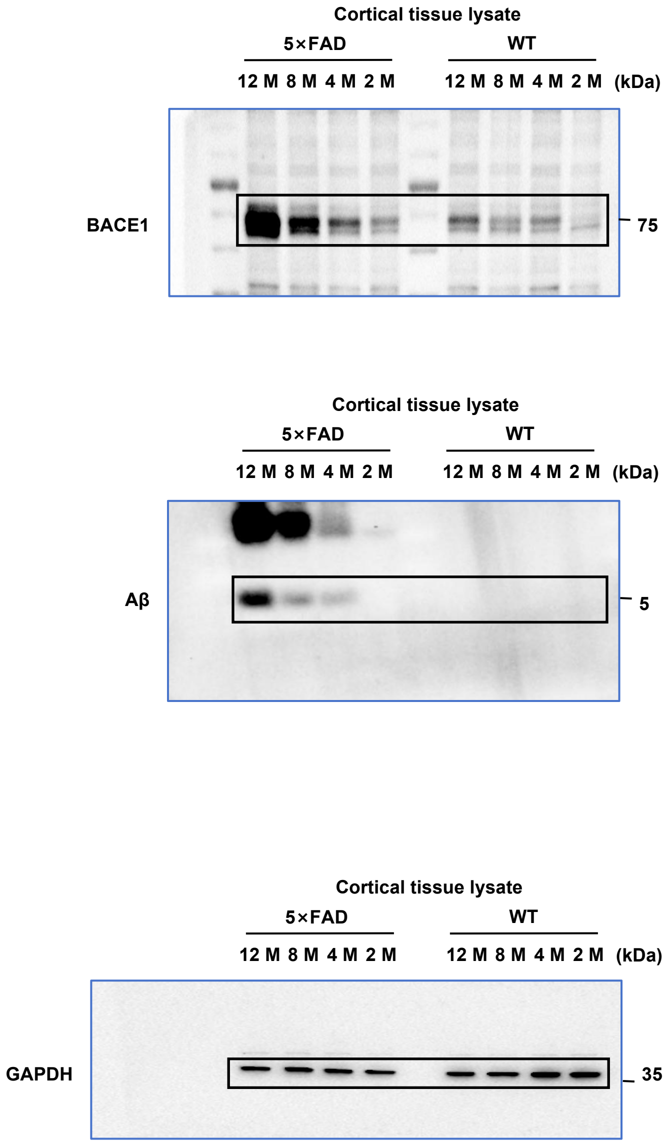

Cortical tissue lysate
5×FAD
WT
12 M 8 M 4 M 2 M 12 M 8 M 4 M 2 M (kDa)
BACE1
75
Cortical tissue lysate
5×FAD
WT
12 M 8 M 4 M 2 M 12 M 8 M 4 M 2 M (kDa)
Aβ
5
Cortical tissue lysate
5×FAD
WT
12 M 8 M 4 M 2 M 12 M 8 M 4 M 2 M (kDa)
GAPDH
35

d

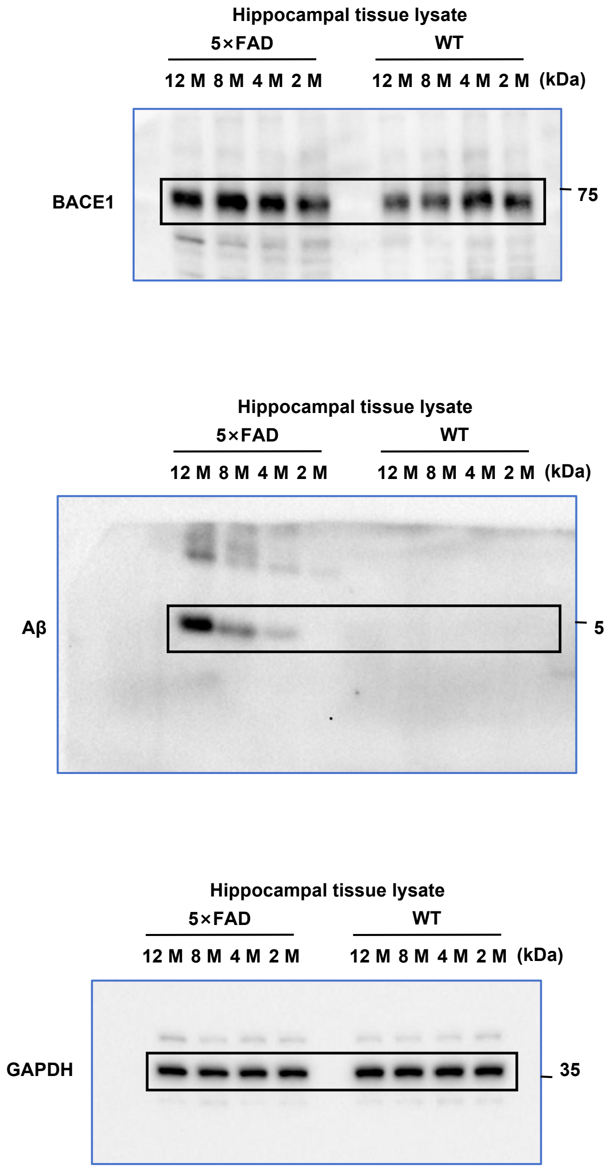

Hippocampal tissue lysate
5×FAD
WT
12 M 8 M 4 M 2 M 12 M 8 M 4 M 2 M (kDa)
BACE1
75
Hippocampal tissue lysate
5×FAD
WT
12 M 8 M 4 M 2 M 12 M 8 M 4 M 2 M (kDa)
Aβ
5
Hippocampal tissue lysate
5×FAD
WT
12 M 8 M 4 M 2 M 12 M 8 M 4 M 2 M (kDa)
GAPDH
35

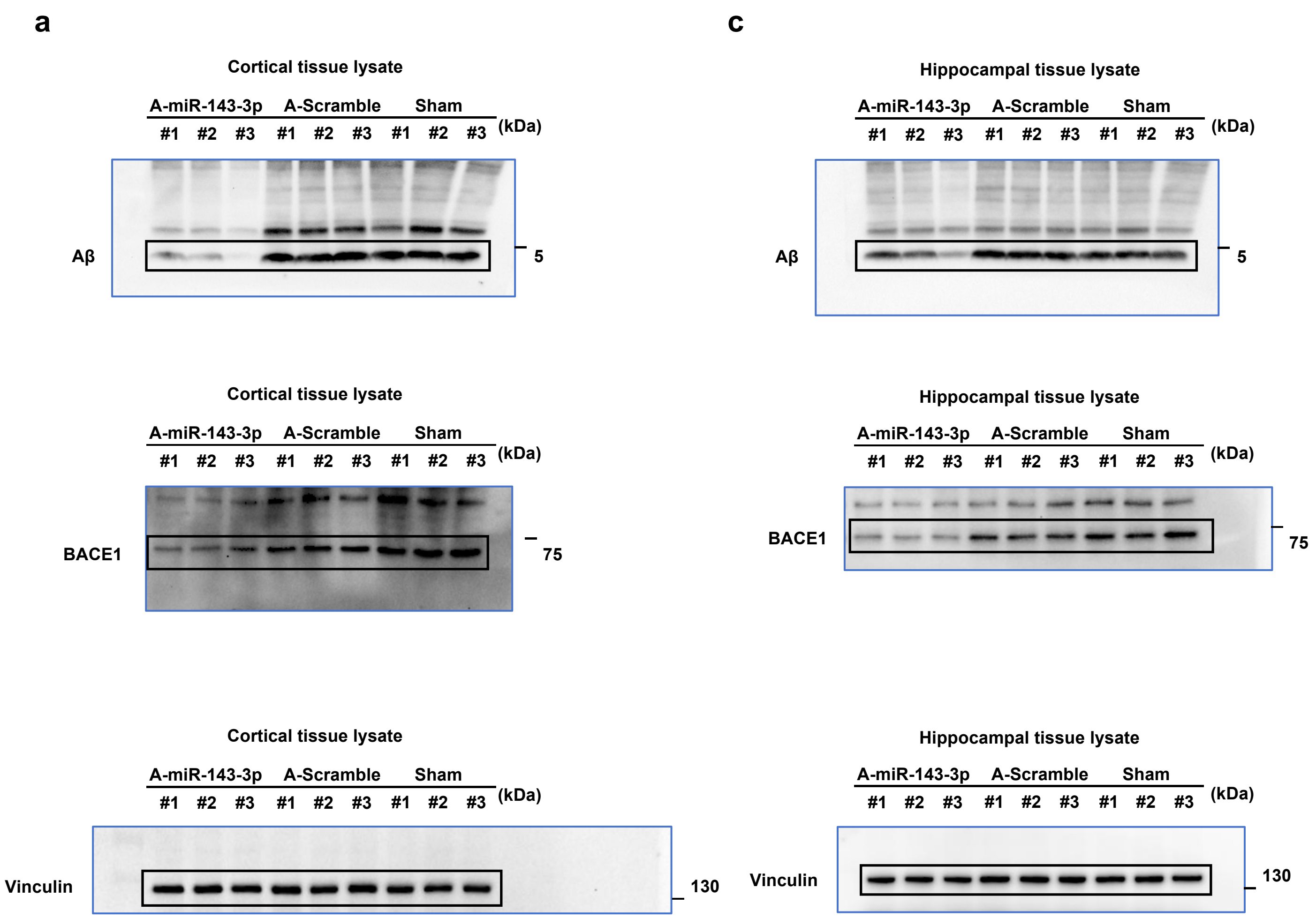
a
Cortical tissue lysate
A-miR-143-3p A-Scramble Sham
#1 #2 #3 #1 #2 #3 #1 #2 #3 (kDa)
Aβ
5
Cortical tissue lysate
A-miR-143-3p A-Scramble Sham
#1 #2 #3 #1 #2 #3 #1 #2 #3 (kDa)
BACE1
75
Cortical tissue lysate
A-miR-143-3p A-Scramble Sham
#1 #2 #3 #1 #2 #3 #1 #2 #3 (kDa)
Vinculin
130
c
Hippocampal tissue lysate
A-miR-143-3p A-Scramble Sham
#1 #2 #3 #1 #2 #3 #1 #2 #3 (kDa)
Aβ
5
Hippocampal tissue lysate
A-miR-143-3p A-Scramble Sham
#1 #2 #3 #1 #2 #3 #1 #2 #3 (kDa)
BACE1
75
Hippocampal tissue lysate
A-miR-143-3p A-Scramble Sham
#1 #2 #3 #1 #2 #3 #1 #2 #3 (kDa)
Vinculin
130

a

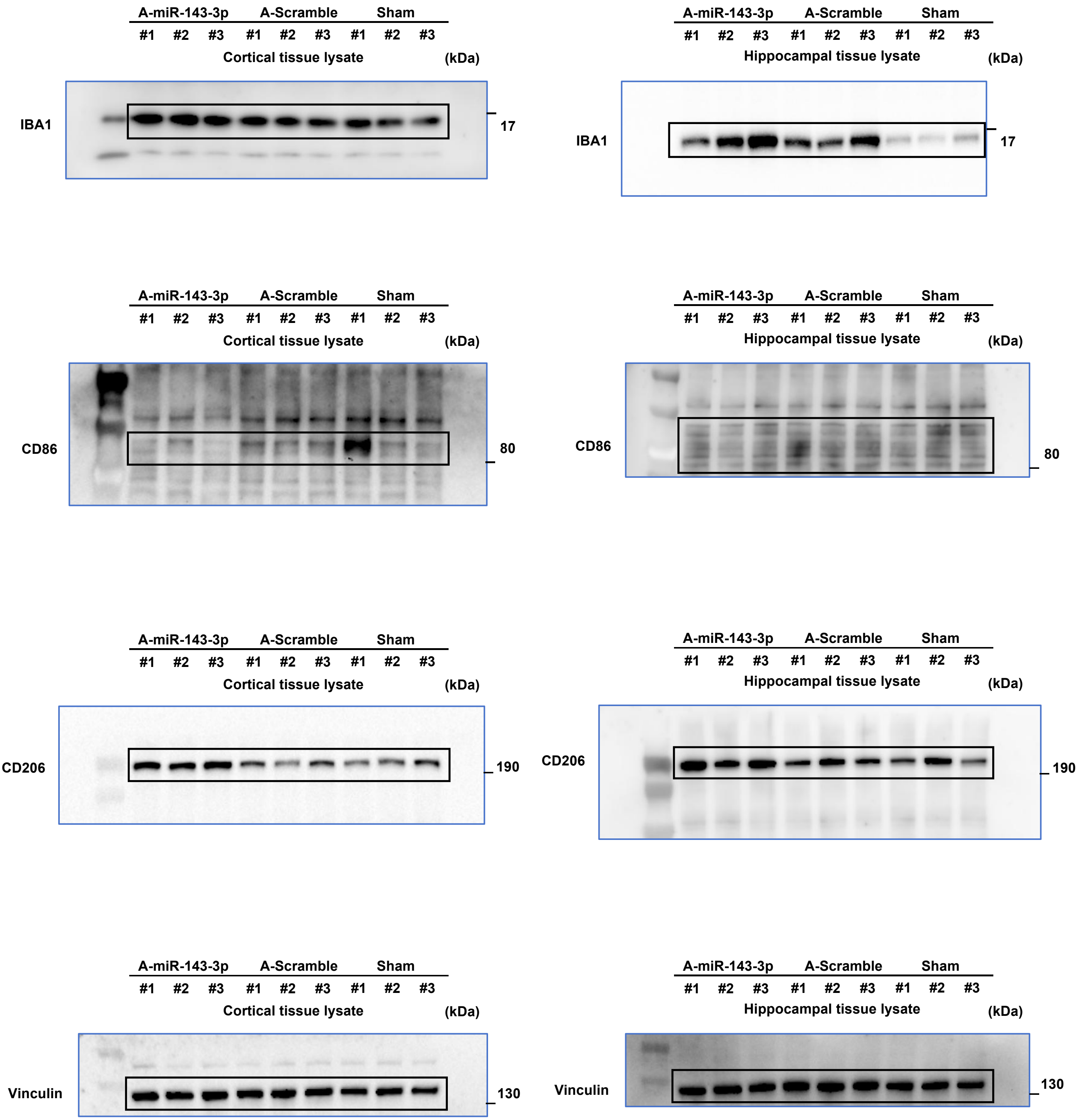
A-miR-143-3p
A-Scramble
Sham
#1 #2 #3 #1 #2 #3 #1 #2 #3
Cortical tissue lysate
Hippocampal tissue lysate
(kDa)
IBA1
17
CD86
80
CD206
190
Vinculin
130

a

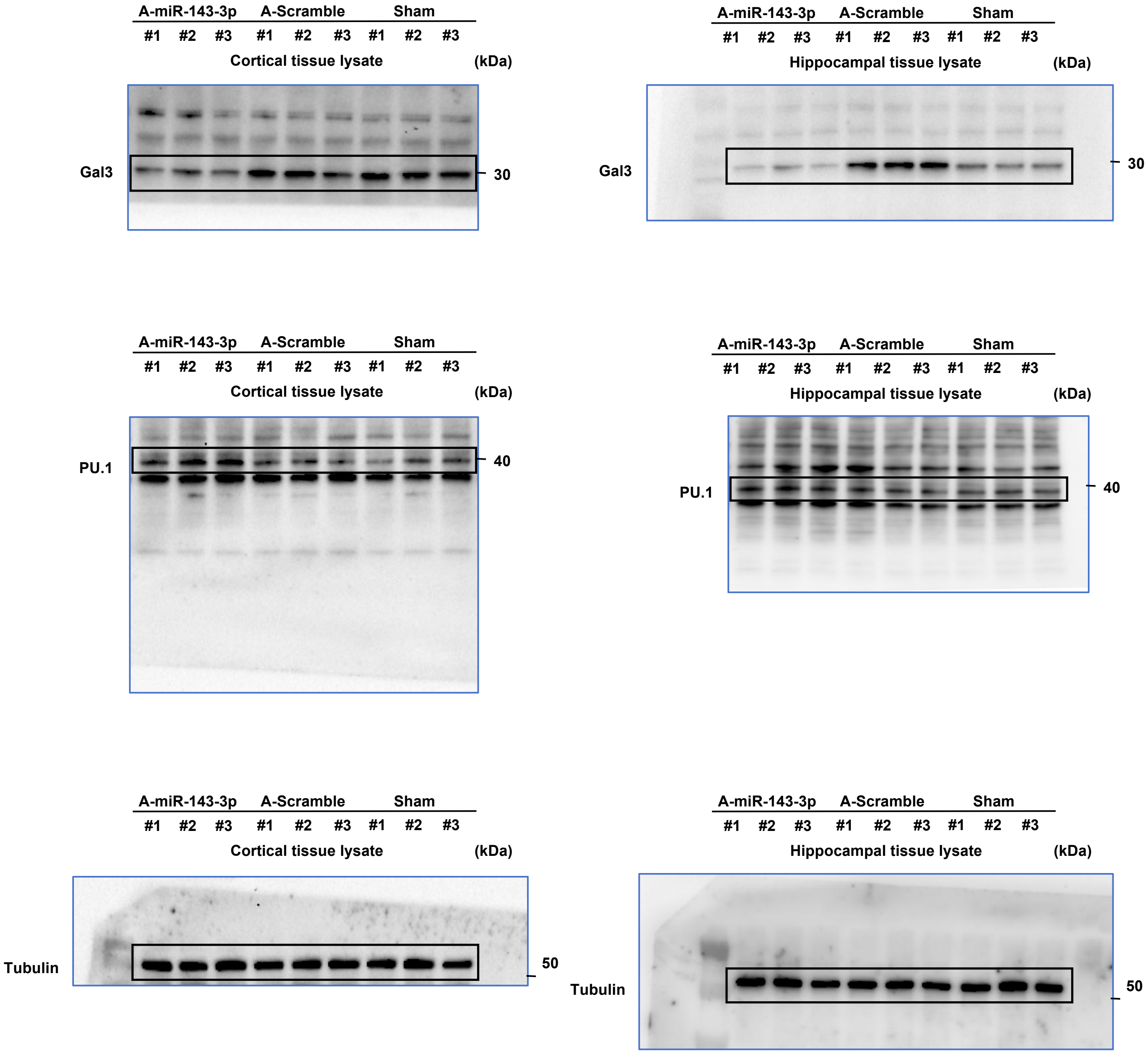
A-miR-143-3p
A-Scramble
Sham
#1 #2 #3 #1 #2 #3 #1 #2 #3
Cortical tissue lysate
(kDa)
Gal3
30
A-miR-143-3p
A-Scramble
Sham
#1 #2 #3 #1 #2 #3 #1 #2 #3
Hippocampal tissue lysate
(kDa)
Gal3
30
A-miR-143-3p
A-Scramble
Sham
#1 #2 #3 #1 #2 #3 #1 #2 #3
Cortical tissue lysate
(kDa)
PU.1
40
A-miR-143-3p
A-Scramble
Sham
#1 #2 #3 #1 #2 #3 #1 #2 #3
Hippocampal tissue lysate
(kDa)
PU.1
40
A-miR-143-3p
A-Scramble
Sham
#1 #2 #3 #1 #2 #3 #1 #2 #3
Cortical tissue lysate
(kDa)
Tubulin
50
A-miR-143-3p
A-Scramble
Sham
#1 #2 #3 #1 #2 #3 #1 #2 #3
Hippocampal tissue lysate
(kDa)
Tubulin
50

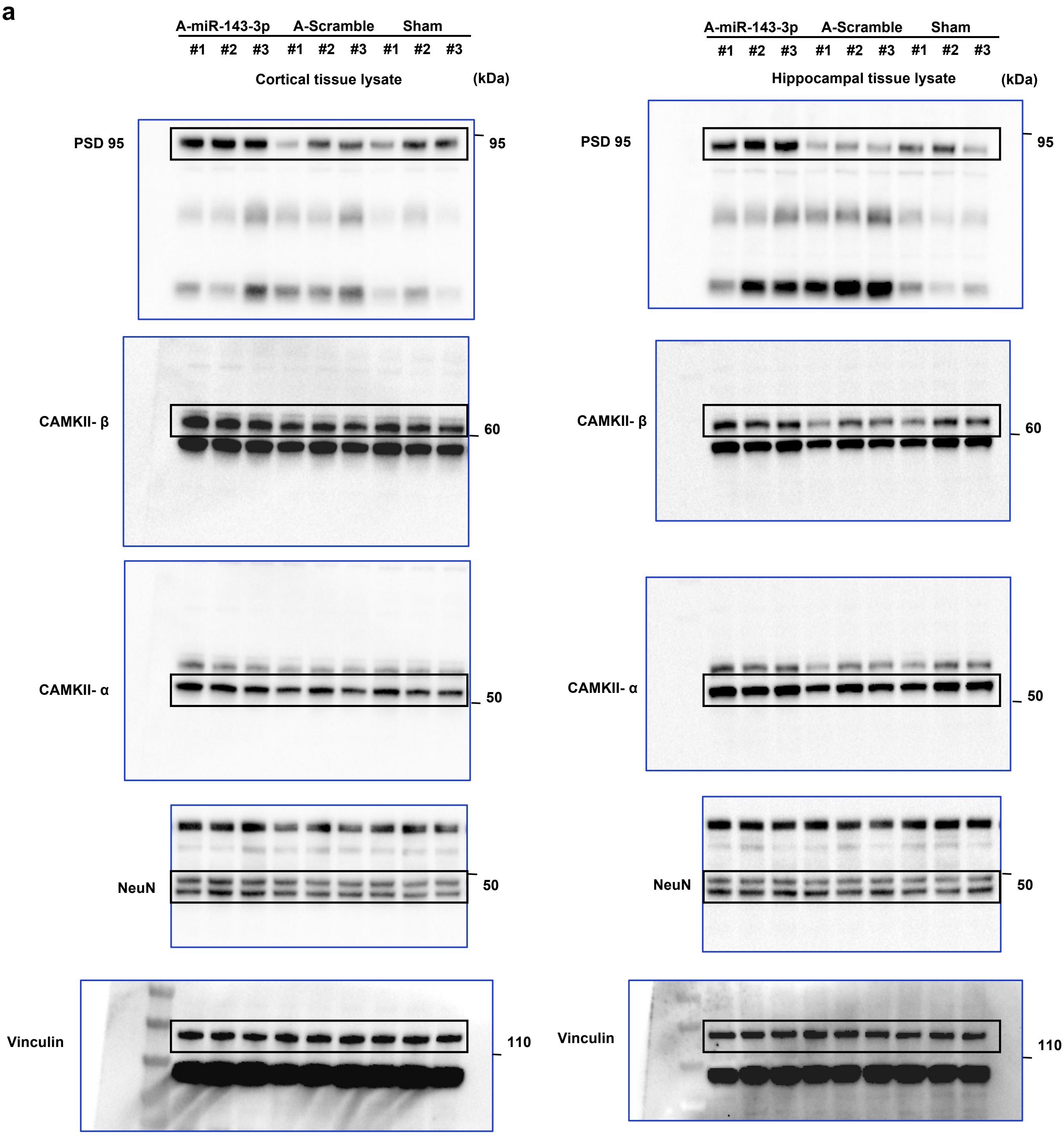
a
A-miR-143-3p
A-Scramble
Sham
#1 #2 #3 #1 #2 #3 #1 #2 #3
Cortical tissue lysate
(kDa)
Hippocampal tissue lysate
PSD 95
95
CAMKII- β
60
CAMKII- α
50
NeuN
50
Vinculin
110

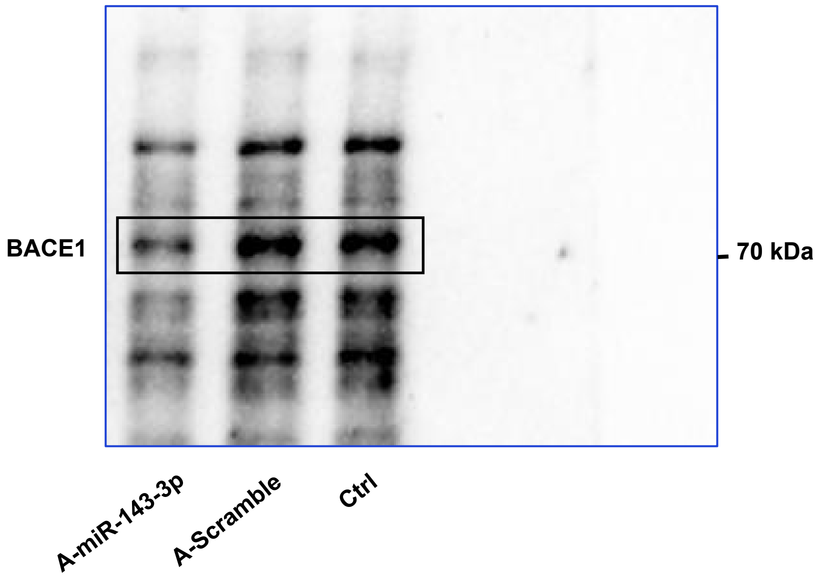
BACE1
70 kDa
A-miR-143-3p
A-Scramble
Ctrl

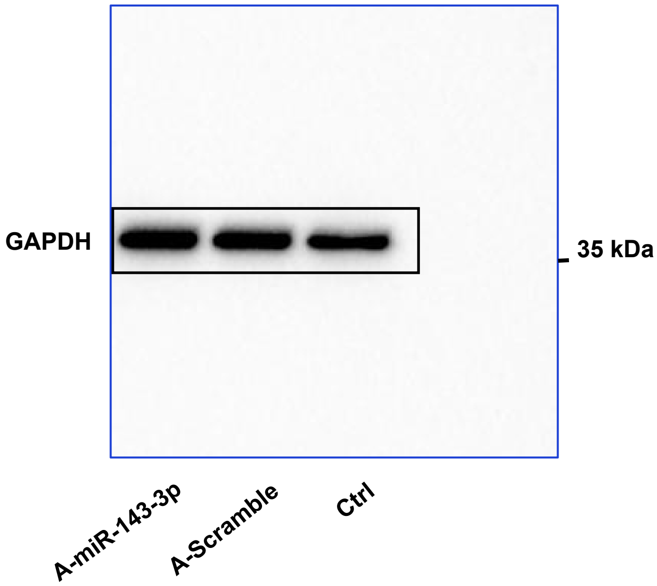
GAPDH
35 kDa
A-miR-143-3p
A-Scramble
Ctrl
